# Genetic Load and Adaptive Potential of a Recovered Avian Species that Narrowly Avoided Extinction

**DOI:** 10.1093/molbev/msad256

**Published:** 2023-11-23

**Authors:** Georgette Femerling, Cock van Oosterhout, Shaohong Feng, Rachel M Bristol, Guojie Zhang, Jim Groombridge, M Thomas P. Gilbert, Hernán E Morales

**Affiliations:** Section for Hologenomics, Globe Institute, University of Copenhagen, Copenhagen, Denmark; Centro de Ciencias Genómicas, Universidad Nacional Autónoma de México, Cuernavaca, México; Department of Human Genetics, McGill University, Montreal, Quebec, Canada; School of Environmental Sciences, University of East Anglia, Norwich, UK; Center for Evolutionary & Organismal Biology, Zhejiang University School of Medicine, Hangzhou, China; Liangzhu Laboratory, Zhejiang University Medical Center, Hangzhou, China; Innovation Center of Yangtze River Delta, Zhejiang University, Jiashan, China; Mahe, Seychelles; Division of Human and Social Sciences, Durrell Institute of Conservation and Ecology, School of Anthropology and Conservation, University of Kent, Canterbury, Kent, CT2 7NR, UK; Center for Evolutionary & Organismal Biology, Zhejiang University School of Medicine, Hangzhou, China; Liangzhu Laboratory, Zhejiang University Medical Center, Hangzhou, China; Innovation Center of Yangtze River Delta, Zhejiang University, Jiashan, China; Division of Human and Social Sciences, Durrell Institute of Conservation and Ecology, School of Anthropology and Conservation, University of Kent, Canterbury, Kent, CT2 7NR, UK; Section for Hologenomics, Globe Institute, University of Copenhagen, Copenhagen, Denmark; University Museum, NTNU, Trondheim, Norway; Section for Hologenomics, Globe Institute, University of Copenhagen, Copenhagen, Denmark

**Keywords:** genetic load, adaptive potential, extinction, conservation, historical genomics

## Abstract

High genetic diversity is a good predictor of long-term population viability, yet some species persevere despite having low genetic diversity. Here we study the genomic erosion of the Seychelles paradise flycatcher (*Terpsiphone corvina*), a species that narrowly avoided extinction after having declined to 28 individuals in the 1960s. The species recovered unassisted to over 250 individuals in the 1990s and was downlisted from Critically Endangered to Vulnerable in the International Union for the Conservation of Nature Red List in 2020. By comparing historical, prebottleneck (130+ years old) and modern genomes, we uncovered a 10-fold loss of genetic diversity. Highly deleterious mutations were partly purged during the bottleneck, but mildly deleterious mutations accumulated. The genome shows signs of historical inbreeding during the bottleneck in the 1960s, but low levels of recent inbreeding after demographic recovery. Computer simulations suggest that the species long-term small N_e_ reduced the masked genetic load and made the species more resilient to inbreeding and extinction. However, the reduction in genetic diversity due to the chronically small N_e_ and the severe bottleneck is likely to have reduced the species adaptive potential to face environmental change, which together with a higher load, compromises its long-term population viability. Thus, small ancestral N_e_ offers short-term bottleneck resilience but hampers long-term adaptability to environmental shifts. In light of rapid global rates of population decline, our work shows that species can continue to suffer the effect of their decline even after recovery, highlighting the importance of considering genomic erosion and computer modeling in conservation assessments.

## Introduction

The global population abundance of 4,392 species monitored over the last 40 decades has declined by 68% (Almond et al. 2022), threatening their long-term viability. On the International Union for the Conservation of Nature (IUCN) Red List, 33,777 species (47.4%) are facing population decline, compared to 36,264 (50.9%) with stable population size, and 1,274 (1.8%) that are increasing in size (IUCN 2022). A growing number of species are being classified as threatened with extinction, i.e. in the Red List categories of Vulnerable, Endangered, or Critically Endangered (Monroe et al. 2019). On the other hand, effective conservation management has been able to recover the population size after a severe bottleneck for a small number of species, resulting in their downlisting on the IUCN Red List (e.g. the snow leopard, the giant panda, and the pink pigeon; (Mallon and Jackson 2017; Swaisgood et al. 2018). However, even when effective conservation actions are capable of reverting population declines, the negative genetic effects that may arise during population declines can persist (Tilman et al. 1994; Kuussaari et al. 2009). Populations that have recovered from a bottleneck could be subjected to a genetic drift debt where they continue to lose genetic diversity, even after demographic recovery (Gilroy et al. 2017; Pinto et al. 2023). Population decline generates genetic drift and inbreeding that erode genetic diversity, compromising the viability of wild populations (Lynch et al. 1995; Lande and Shannon 1996; Willi et al. 2006; Bozzuto et al. 2019). Thus, investigating the evolutionary genomic consequence of population decline in species that have collapsed, but recovered and avoided extinction, improves our understanding of the extinction risk, recovery potential, and the long-term viability of threatened populations.

Empirical and simulation studies have shown that population bottlenecks and long-term small effective population sizes (N_e_) could be conducive to the reduction of deleterious variation through the purging of deleterious mutations (García-Dorado 2012; Hedrick and Garcia-Dorado 2016; Grossen et al. 2020; Dussex et al. 2021; Khan et al. 2021; Kyriazis et al. 2021; Pérez-Pereira et al. 2021; Kleinman-Ruiz et al. 2022; van Oosterhout et al. 2022). Theoretically, this could make species more robust to inbreeding depression. However, small population sizes may also increase the genetic load through the accumulation of mildly deleterious mutations (Grossen et al. 2020; Bertorelle et al. 2022; Smeds and Ellegren 2023). Furthermore, genetic drift in small populations leads to reduced adaptive potential in the face of environmental change (Willi et al. 2006). At present, we have an incomplete understanding of the short- and long-term consequences of population decline and small effective population size on the viability and extinction risk of species (Hedrick and Garcia-Dorado 2016; Mable 2019; Forester et al. 2022).

The rate of genomic erosion and its impact on extinction probability is a complex outcome of the interaction between long-term trends of N_e_, recent population decline, the response of different types of genetic variation (e.g. deleterious mutations and adaptive genetic variation), and the rate of environmental change. Here, we quantify the genomic erosion in the Seychelles paradise flycatcher (*Terpsiphone corvina*), a species whose population declined to 28 individuals in 1965, followed by an (unassisted) recovery to over 250 individuals by the year 2000. Additionally, in 2008 a self-sustaining, growing population was established on Denis Island with translocated individuals. After these demographic gains, the species’ conservation status in the IUCN Red List was downlisted from Critically Endangered to Vulnerable (IUCN 2022/1). We directly compare genomic variation pre- and postpopulation decline by sequencing whole genomes of museum-preserved samples (>130 yr old) and modern samples. We show that the species suffered a 10-fold decline in genome-wide genetic diversity, one of the largest losses compared to other birds with reported historical comparisons. This decline has left the modern Seychelles paradise flycatcher population with a lower genome-wide diversity compared to many other Endangered and Critically Endangered bird species. We used individual-based genomic simulations to investigate how the Seychelles paradise flycatcher managed to avoid extinction after suffering such a drastic population decline and loss of genetic diversity. Our results indicate that the ancestral, prebottleneck population had a low masked genetic load due to their long-term small Ne. This effect was conducive to less inbreeding depression that allowed them to avoid extinction and successfully recover. However, we also show that this long-term small Ne, together with the substantial genetic diversity loss, have likely reduced the species’ adaptive potential and jeopardized their long-term viability when faced with environmental change.

## Results

### Population Structure and Genetic Diversity

**Fig. 1.**
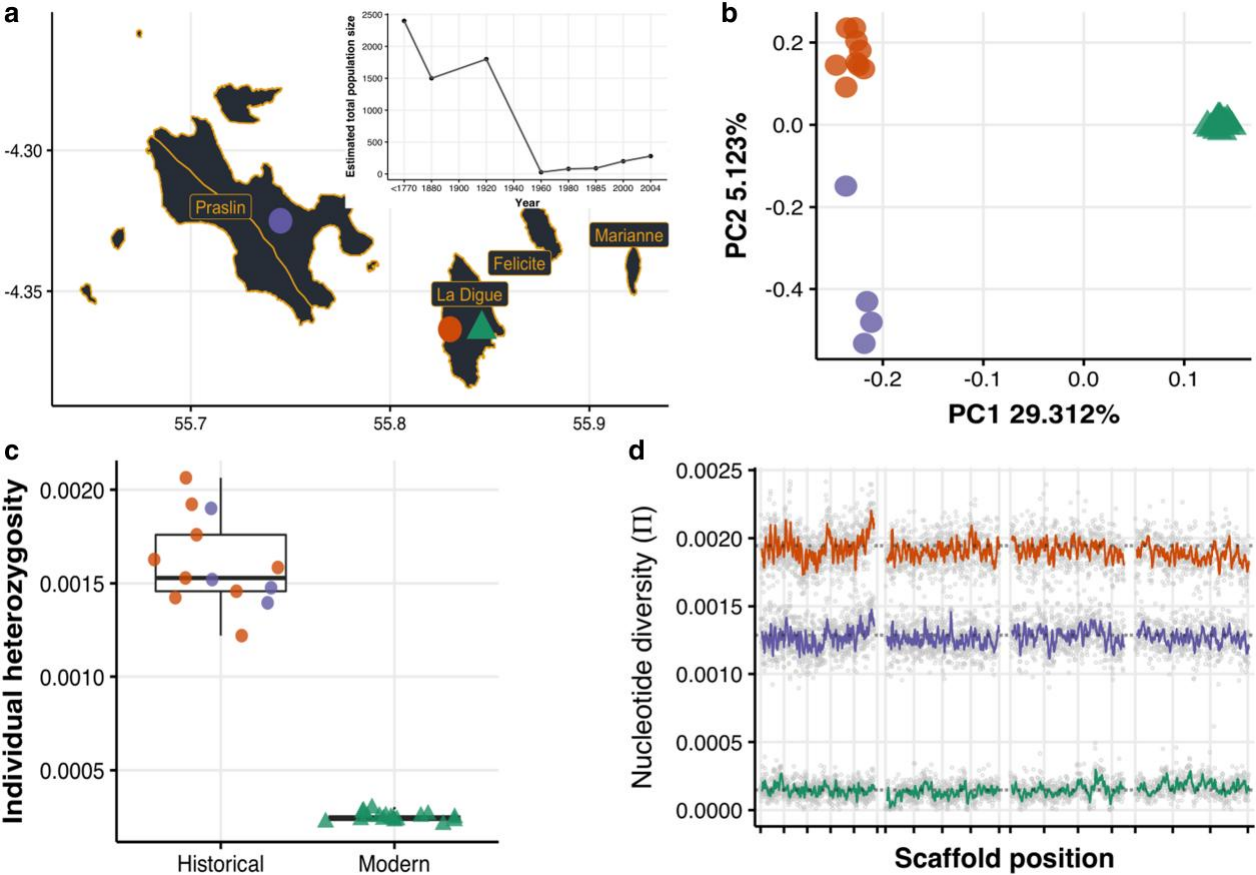
The Seychelles paradise flycatcher shows a massive loss of genome-wide diversity after population decline and despite its demographic recovery. a) Whole-genome sequencing from historical (over 130-yr-old—circles; La Digue and Praslin) and modern (triangles; La Digue) individuals. The inset shows the species’ recent demographic trajectory estimated by (Bristol et al. 2013) showing the dramatic population decline and subsequent recovery. b) Principal component analysis of historical and modern samples. c) On average, modern individuals have 6.4 times less observed heterozygosity than historical individuals. d) On average, the modern population has 10.9 times less nucleotide diversity than the historical populations, and diversity was lost uniformly through the genome (here we show the longest 4 scaffolds; the rest of the scaffolds can be found in supplementary fig. S14, Supplementary Material online). The reason for the lower π in Praslin is that pairwise nucleotide diversity is sensitive to the population sample size and the precision power gained from a larger sample size in La Digue; this effect is not seen in the individual average heterozygosity (panel c).

We analyzed the population genomics of the Seychelles paradise flycatcher, comparing 13 historical samples (coverage: mean = 4.7 sd = 1) and 18 modern samples (coverage: mean = 9.2 sd = 0.4). Historical and modern samples (Fig. 1a) showed a pattern of strong genetic differentiation (PC1; 29% explained variance), with the modern samples forming a homogenous group, and the historical samples differentiated between islands (PC2; 5% explained variance) (Fig. 1b). The rest of the principal components mostly account for the variation within the historical populations (supplementary fig. S1, Supplementary Material online). The admixture analysis assuming three genetic groups (*K* = 3) reflects the strong differentiation between historical and modern individuals and the geographical structure within the historical individuals (supplementary fig. S2, Supplementary Material online). Higher Ks yielded no clear signal of co-ancestry between the historical and modern La Digue individuals. This failure to retrieve a historical component in the modern samples is likely due to strong genetic drift changing the allele frequencies in the modern population (Ebenesersdóttir et al. 2018).

On average, the global individual heterozygosity of modern individuals (La Digue: mean = 0.00024, sd = 0.00002) was 6.4 times lower than that of the historical individuals (La Digue: mean = 0.00162, sd = 0.0003 and Praslin: mean = 0.00157, sd = 0.0002) (Fig. 1c). A genomic sliding-window analysis of population pairwise nucleotide diversity shows that the loss with this metric was 10.9-fold, and that genetic diversity was lost similarly throughout the entire genome (Fig. 1d). Similar comparisons in different bird species have reported smaller losses in nucleotide diversity: the crested ibis and the Chatham Island black robin with a 1.8-fold loss (Feng et al. 2019; von Seth et al. 2022), or in heterozygosity levels: the New Zealand Saddleback, 4.16-fold (Taylor et al. 2007); the Mangrove Finch: 1.32-fold (Lawson et al. 2017); Greater Prairie Chicken: 1.26-fold (Bellinger et al. 2003) (supplementary table S2, Supplementary Material online). The resulting extremely low genetic diversity in the modern population of the Seychelles paradise flycatcher is considerably lower compared to many other threatened bird species (supplementary fig. S3, Supplementary Material online). Our results highlight how even when a population has recovered demographically, it can still be a long way away from recovering in terms of genetic diversity. The results are robust to the difference of depth between modern and historical samples, as all the metrics hold the same pattern when modern samples were down-sampled to the same mean depth of coverage of the historical samples (supplementary figs. S4 to S7, Supplementary Material online). Moreover, ultra-conserved regions of the genome exhibited reduced diversity compared to nonconserved regions in historical samples, but after the population collapses all regions showed a pronounced diversity loss (supplementary fig. S8, Supplementary Material online), providing further evidence of the same extreme effect of the bottleneck. At the same time, the amount of diversity observed in ultra-conserved regions could reflect some moderate effect of DNA damage inflating historical diversity estimates. Therefore, it is important to keep in mind that despite following strict filtering steps and performing several checks (see Methods and supplementary figs. S9 to S13, Supplementary Material online), biases inherit to the analysis of historic DNA cannot be ruled out completely. In particular, the magnitude of diversity loss could be slightly overestimated.

### Demography and Runs of Homozygosity

**Fig. 2.**
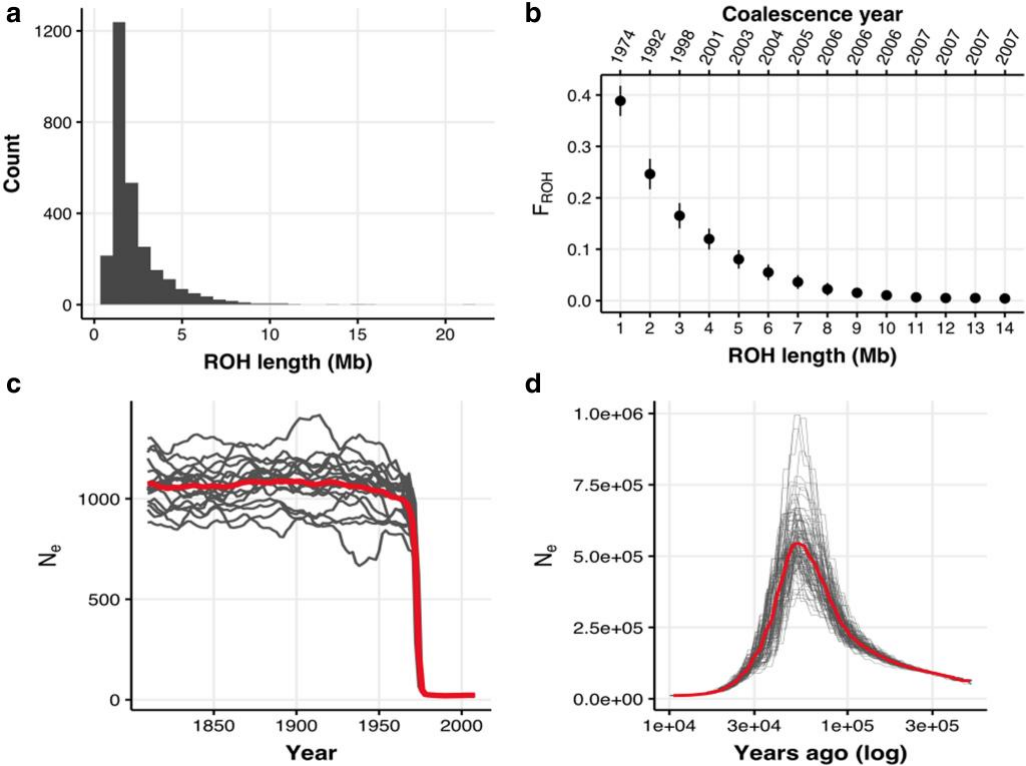
Demographic reconstruction of population decline. a) Runs of homozygosity (ROH) length distribution across all modern individuals. b) Inbreeding coefficient estimated for different classes of ROH lengths (F_ROH_). The year represents the estimated time at which a category of ROH length was formed assuming a recombination rate of 3 cM/Mb and using the formula L = 100/2t cM from (Thompson 2013), where *L* is the ROH length and *cM* is the recombination rate, to obtain *t* the time of ROH coalescence in generations. Generations ago were converted assuming a generation time of 2 yr from the time of sampling. c) Reconstruction of the recent demography (last 100 generations) from LD using GONE (Santiago et al. 2020) assuming a recombination rate of 3 cM/Mb. The mean demographic trajectory accross 40 replicates is shown. Background lines were obtained with a jackknife approach removing one sample at a time. Generations ago were converted assuming a generation time of 2 yr from the time of sampling (2010). d) Reconstruction of the ancient demography (<10,000 yr ago or 5,000 generations ago) from genetic coalescence using the pairwise sequentially Markovian coalescent method (PSMC) (Li and Durbin 2011) assuming a mutation rate of 2.3e-9 and a generation time of 2 yr.

The modern La Digue population has a skewed distribution toward shorter (<5Mb) Runs of Homozygosity (ROHs) (Fig. 2a). Longer ROHs would be expected if closely related individuals mated with each other within the last 10 generations (Fig. 2b; supplementary fig. S15, Supplementary Material online). Hence, the distribution skewed toward shorter ROH length suggests an absence of recent inbreeding in our data (F_ROH_ < 0.01; Fig. 2b). ROHs that are 1 to 2 Mb long (Fig. 2a) are expected to have been formed 10 to 20 generations ago (supplementary fig. S15, Supplementary Material online), which is consistent with historical inbreeding around the year 1974 (F_ROH_ = 0.2 to 0.4), and it is likely to be a product of the bottleneck that started in the mid-1960s (Fig. 2b).

In agreement with the F_ROH_ evidence, the reconstructed recent demographic history (within the last 100 generations) with genetic optimization for *N*_e_ estimation (GONE) (Santiago et al. 2020) also recovered a clear signature of the bottleneck by registering a dramatic drop in the N_e_ around the year 1975 (∼17 generations ago; Fig. 2c), The pairwise sequentially Markovian coalescent method (PSMC) (Li and Durbin 2011) reconstruction shows a very large ancient population that decreased in size and remained small for the last 10,000 yr (Fig. 2d). It is important to note that the deep demographic history reconstruction with PSMC carries some uncertainty. The maximum N_e_ = 530,635 estimated at ∼55,000 yr substantially exceeds the current carrying capacity of the entire Seychelles archipelago. However, past sea levels were highly dynamic, connecting and disconnecting islands in the archipelago on at least 14 separate occasions (Ryan et al. 2009; Warren et al. 2010; Ali 2018). Thus, it is possible that the ancient population could have been much larger at times of increased island connectivity. Seychelles’ landmass is estimated to have been up to 180 times its present size, and gene flow may have been facilitated by islands in the western Indian Ocean that could have acted as stepping-stones between landmasses during the Pliocene and Pleistocene (Cheke and Hume 2008; Warren et al. 2010). This geological signature has been seen in other Seychelles taxa (Groombridge et al. 2002; Rocha et al. 2013; Labisko et al. 2022). However, the large ancestral N_e_ can also be an artifact of population structure, selection, and admixture, all of which are known to introduce biases to coalescent demographic reconstruction (Mazet et al. 2016; Johri et al. 2021; Boitard et al. 2022). For example, if island populations were reproductively separated at some point, PSMC estimates would be inflated as alleles would not coalesce. Irrespective of the uncertainty of ancient N_e_ estimates, we can be confident that the relatively recent genetic lineage remained small for at least 5,000 generations (10,000 yr), in agreement with a history of long-term small N_e_.

### Genetic Load Analyses

**Fig. 3.**
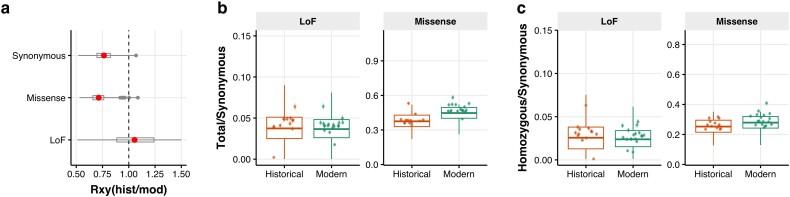
Genetic load dynamics over time. a) Allelic frequency differential between modern and historical samples (Rxy) for 3 categories of putative deleterious mutations. Values equal to one indicate no frequency difference, values below one indicate a higher frequency in the modern population, and values in excess of one a lower frequency in the modern population. Synonymous (mean = 0.71; 95%CI = 0.7–0.72), and missense (mean = 0.76; 95%CI = 0.75–0.77) have significantly increased in frequency in the modern samples (*P* < 0.001), and loss-of-function (LoF) mutations (mean = 1.21; 95%CI = 1.16–1.25) have significantly decreased in frequency (*P* < 0.001). b) Total count of derived alleles in modern and historical individuals for missense and LoF mutation normalized by the count of derived synonymous alleles. There is no significant difference between historical and modern individual counts of LoF alleles (difference = −2.7e-04, 95% CI [−4.4e-04, 9.9e-04], t = 0.75, *P* = 0.45). Modern individuals have a significantly higher count of missense alleles (difference = 0.07, 95% CI [0.07, 0.07], t = 47.7, *P* < 0.001). c) Count of homozygous derived alleles in modern and historical individuals for missense and LoF mutation normalized by the count of derived synonymous alleles. There is a very small, but statistically significant, reduction in the homozygous derived counts of LoF alleles in the modern individuals (difference = −2.2e-03, 95% CI [−2.9e-03, −1.6e-03], t = −6.74, *P* < 0.001). In contrast, modern individuals have a significantly higher count of missense alleles (difference = 0.03, 95% CI [0.02, 0.03], t = 21.32, *P* < 0.001).

We next compared the temporal changes in putative deleterious mutations. Given the massive amount of genetic diversity loss in the modern population (Fig. 2), many deleterious alleles are likely to have been lost due to genetic drift during the bottleneck. However, a few mutations could have drifted to a higher frequency because of less efficient purifying selection in the small-N_e_ population. Therefore, to examine the impact of genetic drift and purifying selection on deleterious variation that remained in the modern population, we conservatively focused on (putative) deleterious alleles that were observed in at least one historical and one modern individual. Mutations classified as synonymous (nearly neutral) and missense (mildly deleterious) exhibited an increased frequency in the modern samples compared to the historical sample, but those classified as loss-of-function (LoF; highly deleterious) exhibited a reduced frequency (Fig. 3a). Next, we counted derived (putatively) deleterious alleles for missense and LoF categories, corrected by the count of derived synonymous alleles. Modern samples showed higher derived counts of missense alleles (Fig. 3b), and also higher counts of homozygous derived missense alleles (Fig. 3c). Although there was no significant change in the counts of LoF alleles (Fig. 3b), the count of homozygous derived LoF alleles went slightly down in the modern samples (Fig. 3c). Altogether, these findings show that severely deleterious (LoF) mutations have been reduced by purifying selection during the bottleneck, although this effect was weak and only affected the load of homozygous LoF mutations. It is important to consider the effect of stringent filtering in temporal analysis involving historic DNA. Only retaining deleterious alleles that were observed both in the historical and modern individuals impaired our ability to detect the full extent of purifying selection on deleterious variation, in particular the effect of purging. While this filtering step is needed to reduce the potential bias caused by sequencing artifacts of historical samples and other sequencing errors, it is a duly conservative approach with a considerable downside. Specifically, it prevented us from finding LoF mutations that are expected to be present at very low frequencies in the ancestral population (Dussex et al. 2023), but which are no longer present in the modern population. Per definition, by using this stringent filtering step, it became technically impossible to detect purging (i.e. the complete removal of harmful variants due to purifying selection). On the other hand, other classes of mutations escaped the effect of purifying selection due to the strong genetic drift, resulting in the increase of nearly neutral (synonymous) and mildly deleterious (missense) mutations.

### Individual-Based Simulations

**Fig. 4.**
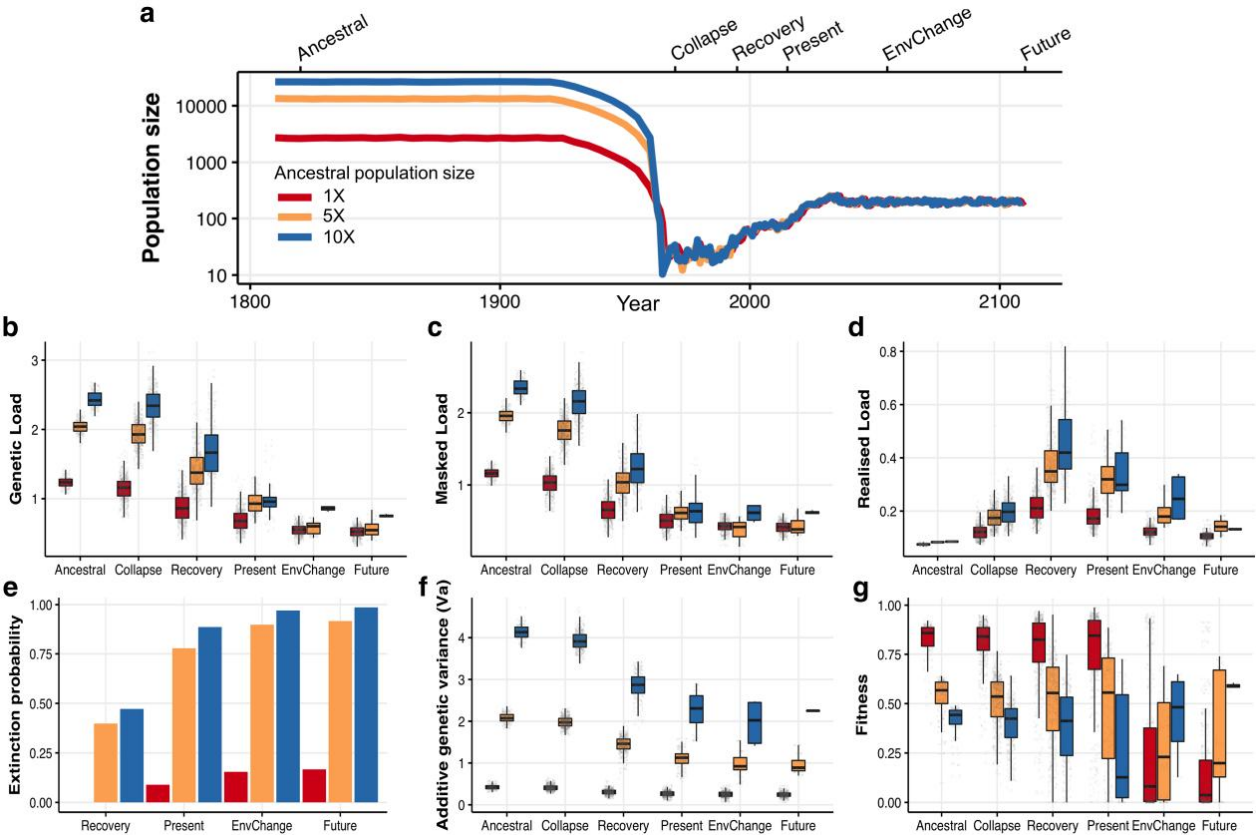
Forward simulations of deleterious and adaptive variation. a) Alternative simulated demographic trajectories. The 1X trend represents the known ancestral size of the Seychelles paradise flycatcher based on the reconstruction of the recent demography with GONE (Fig. 2c). The alternative scenarios represent medium (5X) and large (10X) ancestral population sizes. The trajectory was divided into six stages: Ancestral (years 1810–1815), Collapse (1965–1970), Recovery (1990–1995), Present (2010–2015), Environmental change (2050–2055), Future (2095–2100). During the environmental shift, the quantitative trait optimum value moved from 0.2 to 1.2, resulting in a loss of fitness followed by adaptive evolutionary change. b) Genetic load. c) Masked load. d) Realized load. We calculated the genetic load components, following Bertorelle et al. (2022): $Genetic\;load=\sum_{i=1}^{L}q_{i}s_{i}$, $Realised\;load=\sum_{i=1}^{L}q_{i}^{2}s_{i}+2\sum_{i=1}^{L}q_{i}[1-q_{i}]h_{i}s_{i}$, and masked load = genetic load−realized load. Furthermore, *s_i_* is the selection coefficient, *h_i_* is the dominance coefficient, and *q_i_* is the frequency of the mutation at loci *L*. The genetic load, masked load, and realized load are all in lethal equivalents (see Bertorelle et al. 2022). The reduction in fitness (w) due to the expression of unconditionally deleterious mutations (i.e. inbreeding depression) is a function of the realized load: $w=e^{-\mathrm{Realised}\;\mathrm{Load}}$. e) Extinction probability per scenario (the number of surviving replicates divided by the total number of replicates). f) Additive genetic variance in the quantitative trait. g) Fitness effect conferred by the quantitative trait.

We assessed how different types of genomic variation (deleterious variation and adaptive variation) respond to the population decline and recovery in the species by simulating historical populations with small (1X), medium (5X), and large (10X) ancestral population sizes (Fig. 4a). The total ancestral deleterious variation (i.e. genetic load = sum of masked load plus realized load) scales positively with population size (Fig. 4b). Historically, most deleterious variation is in the form of masked load (Fig. 4c) (i.e. these mutations do not reduce fitness), and only a small proportion is part of the realized load (Fig. 4d) (i.e. mutations that reduce fitness). During the population size collapse, there is a marginal reduction of the genetic load (Fig. 4b) as many rare, low-frequency variants are randomly lost after the bottleneck, and other (mostly high-impact) variants are purged by purifying selection.

On the flip side, during the bottleneck, some of the masked load is converted into realized load by inbreeding (Fig. 4c and d), and this conversion results in a loss of fitness (i.e. inbreeding depression). Two processes are at play here. First, whilst most deleterious variants are lost, genetic drift increases the frequency of a small number of deleterious mutations. Given their now elevated frequency, these deleterious mutations are more likely to be found in homozygous genotypes. Second, the bottleneck increases the probability of mating between closely related individuals. By increasing homozygosity, both genetic drift and inbreeding convert the masked load into a realized load. Figure 4d illustrates this in computer simulations. During the population size collapse, the realized load of the largest ancestral population is increased to around 0.2 lethal equivalents, which equates to a fitness w = e^−0.2^ = 0.82. For the smallest ancestral population size, on the other hand, population size collapse increases the realized load to circa 0.1 lethal equivalents, which equates to w = e^−0.1^ = 0.90. In other words, individuals in large ancestral populations suffer from more severe inbreeding depression during population size collapse than individuals derived from historically small populations (Kyriazis et al. 2021; Mathur and DeWoody 2021; van Oosterhout et al. 2022).

During population recovery, the compositions of the genetic load changed substantially (Fig. 4b–d). After having experienced the effect of the bottleneck for longer, the realized load peaked at over 0.4 lethal equivalents for the largest ancestral population size. Severe inbreeding depression during this stage would have reduced the fitness of individuals markedly, w = e^−0.4^ = 0.67 (i.e. 33% individual fitness loss on average). Figure 4d shows that the worst affected individuals express a realized load of 0.8 lethal equivalents, which means that their fitness would be less than half that of their prebottleneck ancestors. In contrast, the smallest ancestral population (i.e. the simulations most similar to the Seychelles paradise flycatcher's historical demography) suffer much less inbreeding depression at this point. An average individual is expected to express 0.2 lethal equivalents, a ∼18% reduction in fitness. This explains why the small population has a lower extinction risk (Fig. 4e), and why the Seychelles paradise flycatcher may have avoided extinction. After recovery, natural selection regains power in an expanding population and the realized load is once more selected against, reducing the genetic load (Fig. 4d).

In the same models, we also simulated adaptive variation as additive genetic variants. Unlike unconditionally deleterious mutations (i.e. the genetic load) that always reduce fitness when expressed, additive genetic variants can either increase or decrease fitness depending on the genetic background and the environment (Charlesworth 2013a, 2013b). Figure 4f shows that the amount of additive genetic variation (V_a_) increases with the ancestral population size. In a stable environment, ancestrally larger populations have on average lower fitness (Fig. 4g). This is because they contain more segregating variants which can produce more extreme (i.e. suboptimal) phenotypes (Charlesworth 2013b). However, their larger V_a_ gives them a wider phenotypic breath and a greater adaptive potential when environmental conditions change. As expected, population size collapse reduces V_a_, but as with the genetic load, the effect on quantitative genetic variation is most pronounced during recovery (Fig. 4f). Remarkably, the loss in V_a_ results in the most pronounced fitness loss in large ancestral populations. However, after environmental change, recovered populations derived from large ancestral populations can better match the new environmental optimum. Their superior adaptive potential ensures that such populations have a higher fitness during environmental change in the future (Fig. 4g).

## Discussion

We analyzed the whole genome sequence data of a threatened species that suffered a population decline to 28 individuals, the Seychelles Paradise flycatcher (*Terpsiphone corvina*), comparing the level of genomic erosion between 13 historic (>130-yrs-old) and 18 modern birds. In addition, we conducted computer simulations to study the effects of population decline and recovery on the genetic load and adaptive evolutionary potential. We thus assessed the long-term impacts of changes in genomic variation on population viability. We uncovered a 10-fold loss of genetic diversity in the Seychelles paradise flycatcher, reflecting severe genetic drift during population size decline that continues to act despite population recovery. We also found evidence of historical inbreeding at the time of population decline, but no evidence of recent inbreeding, reflecting successful population recovery. Demographic reconstructions suggest that prior to its recent population decline, the Seychelles paradise flycatcher sustained a small effective population size (N_e_) for thousands of generations. Our genomic simulations suggest that this reduced the amount of (masked) genetic load in the ancestral population, resulting in only mild inbreeding depression during its collapse. In other words, the long-term small N_e_ of this species may have allowed for its (unassisted) demographic recovery and helped avoid extinction. However, the species has not recovered its genetic diversity, and the mean fitness of individuals is predicted to be lower than that of their ancestors. Our simulations also indicate that the loss of genetic diversity has likely reduced their adaptive potential, and this reduction could jeopardize the species’ long-term viability when faced with environmental change. Our analyses illustrate the power of historical versus modern comparisons, in combination with analyses of genomic erosion and simulations to assess the medium to long-term effects of population decline and recovery on population viability (Díez-del-Molino et al. 2018; Feng et al. 2019; Dussex et al. 2021; Sánchez-Barreiro et al. 2021). Importantly, we showcase how to use this integrative approach to inform conservation assessments (Jensen et al. 2022; van Oosterhout et al. 2022).

### Historical Inbreeding, But Not Recent Inbreeding

Remarkably, we did not find evidence of long ROH, which is typically observed in recently bottlenecked species such as the crested Ibis (Feng et al. 2019), the alpine ibex (Grossen et al. 2020), the white rhinoceros (Sánchez-Barreiro et al. 2021), and different horse breeds (Grilz-Seger et al. 2018). Instead, we found that the most common category of ROHs was between 1 to 2 Mb long. ROHs are formed when closely related individuals mate (i.e. consanguineous mating or inbreeding). The inbred offspring inherit identical segments of DNA, which show up as ROH across the genome. If this offspring mates with unrelated individuals in later generations, the ROHs can be “broken down” by recombination and they become shorter. Thus, the distribution of ROH size reflects an inbreeding timeline. Our results suggest that the population decline imposed severe inbreeding and that at the time of the bottleneck individuals had ∼40% of their genomes contained in ROHs (F_ROH_ = 0.4; Fig. 2b). Upon demographic recovery, ROHs were broken down by recombination, leaving this signature of relatively shorter ROHs (1 to 2 Mb), consistent with historical inbreeding (∼45 yr ago).

The lack of long ROHs, on the other hand, indicates the absence of recent inbreeding (F_ROH_ < 0.01 in the last decade; Fig. 2b), meaning that the demographic recovery allowed the Seychelles paradise flycatcher to avoid consanguineous mating. Even though pairs of Seychelles paradise flycatchers normally retain the same territory for life and are socially monogamous, their rate of extra-pair paternity is very high, with 71% of chicks being the biological offspring of males from different territories across the island (Bristol 2014). This could explain why once the population recovered demographically it was able to avoid inbreeding.

### Genetic Load, Adaptive Potential, and Extinction Risk

Consistent with theoretical expectations (Hedrick and Garcia-Dorado 2016; Santiago et al. 2020; Bertorelle et al. 2022; Dussex et al. 2023), and empirical observations in other taxa (Grossen et al. 2020; Dussex et al. 2021; Khan et al. 2021; Pérez-Pereira et al. 2021; Kleinman-Ruiz et al. 2022; Robinson et al. 2022), we observed a distinct pattern for highly deleterious and mildly deleterious variation after the bottleneck. It is important to note that we focused solely on deleterious alleles that remained in the modern population. This minimized the bias caused by sequencing artifacts typical in the analysis of historical samples. Furthermore, many deleterious variants have been removed during the bottleneck, and we are interested in analyzing the fate of the remaining genetic load of segregating mutations. Theory predicts that population bottlenecks reduce the number of segregating sites with deleterious mutations, but also that they increase the frequency of deleterious variants at some loci that survive the bottleneck (Dussex et al. 2023). This elevated frequency increases the level of homozygosity, which increases the realized load and leads to inbreeding depression (Hedrick and Garcia-Dorado 2016; Santiago et al. 2020; Bertorelle et al. 2022). In turn, this allows for purifying selection to reduce some of the realized load. We observed only marginal evidence for purifying selection on highly deleterious variants, i.e. the loss-of-function (LoF) mutations. The genetic load of homozygous LoF mutations decreased slightly after the bottleneck. These variants have the strongest fitness effects and are thus most effectively removed by selection during inbreeding. On the other hand, derived alleles of mildly deleterious variants (i.e. missense mutations) increased in frequency and count. This is because purifying selection is less efficient in populations with a small effective size. During population decline, inbreeding and genetic drift convert the masked load into realized load (e.g. Mathur and DeWoody 2021; Smeds and Ellegren 2022). Because of the reduced efficacy of purifying selection, a portion of the converted load escaped selection and persisted as realized load, reducing population viability (Grossen et al. 2020; van Oosterhout et al. 2022; Pinto et al. 2023).

Small-island species with long-term small N_e_ accumulate less masked load compared to mainland species with large ancestral N_e_. Such species will have less segregating masked genetic load to be converted into realized load during a bottleneck, particularly from highly deleterious variants (Dussex et al. 2023). Therefore, this could make small-island species more resilient to the effects of strong inbreeding depression during population decline, although mild inbreeding depression could still operate. Possibly this could also explain why we observe a modest effect of purifying selection on the LoF variation. Our reconstruction of the recent demographic history of the Seychelles paradise flycatcher is consistent with a scenario in which prior to the recent bottleneck of 1964, the species had a long-term small N_e_ for the last ∼5,000 generations. Given that historically large populations possess a high masked load, they are particularly prone to the detrimental effects of load conversion during population size collapse (Mathur and DeWoody 2021; van Oosterhout et al. 2022). Conversely, the Seychelles paradise flycatcher was particularly resilient to inbreeding depression and this likely played a role in their successful (unassisted) demographic recovery. This hypothesis of long-term reduction of the genetic load in the Seychelles paradise flycatcher is consistent with the observed signal of historical population structure between islands, also observed by Bristol et al. (2013), which used microsatellites for more historical samples distributed across multiple islands. This could have been conducive to effective long-term reduction of deleterious variation as selection operated on small ancestral populations with little interisland gene-flow.

The Seychelles paradise flycatcher population size has been steadily increasing in the past 20 yr. Nonetheless, even after the apparent demographic recovery, the modern population possesses a very low genetic diversity. This is of conservation concern because genome-wide diversity is an important predictor of population fitness and adaptive potential (Hansson and Westerberg 2002; Fagan and Holmes 2006; Willi et al. 2006, 2022; Harrisson et al. 2014; Willoughby et al. 2015; Kardos et al. 2021; Mathur and DeWoody 2021). Our computer simulations show the loss of genetic diversity may lead to reduced adaptive response during environmental change. In turn, and opposite to the prediction for the genetic load, this could elevate its extinction risk compared to populations with a larger ancestral N_e_ (Lande and Shannon 1996; Willi et al. 2006). In summary, the long-term small ancestral N_e_ represents a tradeoff in which populations might be more resilient in the short-term when facing strong bottlenecks, but less resilient in the long-term in the face of environmental change.

### The Role of Genomics in Species Conservation Assessments

The incorporation of genetic information into assessments of conservation status and policy remains inadequate (Hoban et al. 2020; Laikre et al. 2020). Here, we show the impact of genomic erosion in the Seychelles paradise flycatcher, a species that has made a successful demographic recovery that resulted in its downlisting in the IUCN Red List of Threatened Species. Our findings suggest that its ancestrally small N_e_ might have conferred resilience to inbreeding that initially eases demographic recovery. However, it may also compromise its adaptive potential, particularly during environmental change. Moreover, it is important to note that the reduction of their ancestral genetic load happened (naturally) over thousands of generations. In addition, the chronic reduction in fitness caused by an elevated realized load is likely to put the species at increased risk of extinction. This might be of particular relevance to other island endemic species, which are, for example, characterized by reduced immune function, partially due to their low N_e_ (Barthe et al. 2022). Accordingly, low genetic diversity can make species more prone to emerging infectious diseases and interspecific competition, which is a substantial risk given the high rates of new colonisations and invasive species in islands (Sax and Gaines 2008; Lockwood et al. 2009). Our work demonstrates the power of direct comparisons between historical and modern whole genomes to reconstruct the temporal dynamics of diversity, demography, and inbreeding, and the importance of combining these insights with simulations to inform conservation. We argue that the downlisting of the IUCN Red List status may sometimes be premature and species assessments should include an assessment of the risks posed by genomic erosion. A promising way forward to achieve this is incorporating the analysis of genomic erosion in population viability analysis (PVA) with novel computer simulations methods to leverage the full power of genomic and ecological/demographic data.

## Methods

### Study System and Sampling

The Seychelles paradise flycatcher historically inhabited five islands in the Seychelles archipelago. In the early 1900s, the species disappeared from three islands (Aride, Felicité, and Marianne), and in the 1980s disappeared from a fourth one (Praslin). Restricted to a single island (La Digue) in 1965 the population size was reduced to 28 individuals. By the year 2000, the population recovered to ∼250 individuals, relatively unassisted. In the year 2008, 23 individuals from La Digue were introduced to Denis Island and successfully established (Henriette and Laboudallon 2011). These populations continue to grow without assistance, with a current estimated species census population size of 350 to 506 individuals (IUCN 2022/1).

A previous study of historical versus modern diversity using 14 microsatellites reported a significant reduction in heterozygosity after the bottleneck (Bristol et al. 2013). Following Bristol et al. (2013), we sampled 13 historical individuals collected between 1877 to 1888, and 19 modern individuals collected between 2007 and 2008 (supplementary table S1, Supplementary Material online; Fig. 1a). Historical samples were sourced from natural history collections as small (2 to 4 mm) pieces of toe-pad. Information about preservation methods is generally not available for old samples, however, bird toe-pads are often a good source of endogenous DNA. This is because their preservation method, which normally involves natural drying, is less harmful compared to the arsenic and formalin treatments commonly used elsewhere (Tsai et al. 2020).

### Genomic Libraries and Sequencing

Historical DNA extractions were carried out with a modified version of Campos and Gilbert (2012) in a polymerase chain reaction (PCR-)free clean laboratory exclusively designated for ancient DNA. Historical single-stranded sequencing libraries were prepared following the Santa Cruz Reaction protocol (Kapp et al. 2021), as modified for the DNBSEQ-G400 sequencing platform (van Grouw et al. in review), and amplified in three indexed PCR reactions. Modern DNA extractions were done with the DNAeasy commercial kit (Qiagen) following the manufacturer's recommendations and directly submitted to BGI Copenhagen for sequencing in their DNBSEQ-G400 platform.

### Sample Processing

We assessed the quality of the raw sequencing reads by running FastQC (Andrews 2010) and summarized the results with MultiQC (Ewels et al. 2016). We then mapped the sequencing reads to the publicly available de novo sequenced reference genome of the Seychelles paradise flycatcher produced by the B10K consortium (Zhang et al. 2015), available at https://b10k.scifeon.cloud/#/b10k/Sample/S15237). We ran the automated pipeline PALEOMIX (Schubert et al. 2014) per sample for both the historical and the modern datasets. This pipeline carries out the preprocessing steps of removing adapters and collapsing mate reads, read mapping with BWA (Li and Durbin 2010), and quantifies the postmortem damage of historical samples by running MapDamage (Ginolhac et al. 2011) (see supplementary figs. S16 and S17, Supplementary Material online for results). We employed the *aln* algorithm with default parameters for mapping historical samples ignoring reads shorter than 30 bp. This algorithm has shown good performance for short and damaged reads (Schubert et al. 2012), moreover, this is the recommended BWA algorithm for reads shorter than 70 bp (Li and Durbin 2010). We employed the *mem* algorithm for modern samples as it is the recommended BWA algorithm for reads longer than 70 bp and represents considerable gains in speed (Li and Durbin 2010). Duplicates were removed with picard MarkDuplicates (Broad Institute 2018) and InDels were realigned using the tools IndelRealigner and RealignerTargetCreator available in GATKv3.5 (Van der Auwera and O’Connor 2020). We estimated the percentage of endogenous content by computing the rate between uniquely mapped reads and total reads. On average, historical samples had an endogenous content of 57.26% (sd. 4.00%), and modern samples of 96.23% (sd.1.86%; supplementary table S1, Supplementary Material online). A summary of additional read and mapping statistics, as well as sample metadata, can be found in supplementary table S1, Supplementary Material online.

We performed a depth-based analysis to identify and remove sex chromosome-derived scaffolds following Pečnerová et al. (2021) as their sex-biased pattern of inheritance can bias genetic diversity estimates. Considering that females are the heterogametic sex in birds (ZW), we calculated the difference in the normalized average depth between males and females, expecting that coverage will be nearly double in males relative to females for the Z chromosome, and absent in males but present in females for the W chromosome. In total, 1,308 potential sex-linked scaffolds were removed from the subsequent analyses (73.4 Mb putatively Z-linked and 10.7 Mb putatively W-linked).

The small population size and population structuring can result in the sampling of closely related individuals which would inflate the estimated level of inbreeding. To avoid this, we identified and removed closely related individuals using NGSrelate2 (Hanghøj et al. 2019). We used a threshold of KING-robust kinship ≥ 0.25, R0 ≤ 0.1, and R1 ≥ 0.5 as described by Waples et al. (2019). We found only one closely related pair in the modern dataset, and we removed one of these individuals from the final dataset (supplementary fig. S18, Supplementary Material online). The pedigree metadata confirmed these two individuals had a parent-offspring relationship.

### Historical DNA Biases

Historical DNA is subject to postmortem DNA damage and contamination. This commonly leads to short sequencing reads that are error-prone and have lower quality, and samples that have low endogenous content and a low depth of coverage. We took several steps to counteract these challenges. First, we confirmed that two common features of ancient DNA datasets: reduced average sequence lengths and low coverage, did not generate reference genome mapping biases (Gopalakrishnan et al. 2022) in our historical dataset (supplementary fig. S9, Supplementary Material online). Second, we used dedicated software for low-coverage samples, ANGSD 0.921 (Korneliussen et al. 2014), to estimate genotype likelihoods and avoid directly calling genotypes. Across all ANGSD methods, we used the GATK algorithm, filtered for a base quality of 20 and a mapping quality of 30. For each population or group of samples, we computed the 1% and 99% quantiles of global depth to filter out regions with extremely low and extremely high depth. For single-nucleotide polymorphism (SNP) calling we inferred the major and minor alleles and used the likelihood test with a *P*-value threshold of 1 × 10^−6^. Third, as commonly done in ancient DNA analyses to counteract biases from postmortem DNA damage, we removed transitions for all analyses that compared historical and modern samples. Finally, we confirmed that our finding that the modern population lost a considerable part of its genetic diversity was not heavily biased by the low quality of historical reads by comparing the loss of diversity across the genome (supplementary fig. S10, Supplementary Material online) against mapping quality (supplementary fig. S11, Supplementary Material online), average depth (supplementary fig. S12, Supplementary Material online), and DNA damage (supplementary fig. S13, Supplementary Material online).

### Population Structure and Genetic Diversity

We performed a Principal component Analysis with PCAngsd 1.01 (Meisner and Albrechtsen 2018) with the genotype likelihoods of the joint historical-modern dataset. Next, we estimated their admixture proportions with NGSAdmix (Skotte et al. 2013), running 250 independent runs from *K* = 2 to *K* = 6. We evaluated the different runs using EvalAdmix (Garcia-Erill and Albrechtsen 2020) and estimated the best K using Clumpak Best K algorithm (Kopelman et al. 2015). We visualized the proportions using PONG (Behr et al. 2016).

Per-sample global heterozygosity estimates were computed directly from the site frequency spectrum (SFS) of each sample by calculating the genome-wide proportion of heterozygous genotypes. We first computed the site allele frequency (SAF) per sample in ANGSD, followed by the realSFS function to get the folded SFS assuming the reference genome as the ancestral state. We bootstrapped the SFS estimation 300 times.

To estimate the genome-wide nucleotide diversity (π) we first estimated the population-level folded SFS as done with the heterozygosity analysis but providing as input all the samples per group. We calculated per site π directly from each population's SFS in two steps following the approach of Korneliussen et al. (2013) by dividing the pairwise Watterson theta value (Watterson 1975; Dung et al. 2019) over the effective number of sites with data (i.e. including all nonvariable sites that passed the filters) per window. We computed these statistics using nonoverlapping sliding windows of 50 Kb.

### Demography and Runs of Homozygosity

Genotypes were called with ANGSD from the genotype likelihoods as described above to identify ROH in modern individuals with PLINK v1.9 (Purcell et al. 2007). SNPs not in Hardy–Weinberg equilibrium were removed and the remainder SNPs were pruned based on linkage disequilibrium (LD) *r*^2^ > 0.8 as implemented in Foote et al. (2021). The following parameters were used to estimate ROHs: minimum window size = 10 SNPs, minimum density per 50 kb = 1 SNP, maximum heterozygous sites per window = 5, and a maximum distance between SNPs = 1000 kb.

Analysis of recent (<100 generations) demography was performed with GONE (Santiago et al. 2020) which uses the patterns of LD to estimate recent population size changes. We used unphased genotypes of the modern samples as described for the ROHs and assumed a recombination rate of 3 cM/Mb with 40 replicates and default parameters. In order to have an estimate of the bias and variance of the results, we did a jackknife cross-validation by sampling out one individual at a time and computing the demography with GONE at each iteration. No subsampling of SNPs was needed as none of the 148 used scaffolds exceeded the 100,000 upper limit of GONE. A total of 1,368,272 genome-wide SNPs were used in each iteration.

Long-term (>5,000 generations) demography analysis was calculated with PSMC (Li and Durbin 2011) using the publicly available reference genome that was sequenced to a depth of coverage of 75x. The consensus diploid sequence was computed using SAMTOOLS and bcftools (Danecek et al. 2021). The settings for the PSMC were as follows: -N30 -t5 -r5 -p “4 + 30*2 + 4 + 6 + 10” following Nadachowska-Brzyska et al. (2015). A total of 100 independent bootstrap rounds were combined and the final plot was generated assuming a mutation rate of 4.6e-9 (as reported in the collared flycatcher; Smeds et al. 2016) and a generation time of 2 yr (R. Bristol 2014, unpublished data).

### Genetic Load Analyses

We individually called high-quality SNPs in each of the historical and modern individuals with bcftools (Danecek et al. 2021) to produce a gvcf file (i.e. including invariant sites), retaining all sites with a minimum base and mapping quality of 30, a minimum depth of 4X and a maximum of 34X, and ignoring InDels and their surrounding SNPs (5 bp). We individually annotated each filtered SNP file with SNPeff v.4.3. (Cingolani et al. 2012) using a custom database with our annotated reference genome. We classified putatively deleterious variants into three categories (i) Low-impact variants that are likely to be not deleterious (i.e. synonymous), (ii) Moderate-impact variants that are likely to modify the protein effectiveness (i.e. missense), and (iii) High-impact variants are likely to disrupt the protein function (i.e. loss of function LoF) (Cingolani et al. 2012). We merged the annotated gvcf files and retained only variants with less than 30% missing data and whose derived alleles were present in at least one individual of each of the historical and modern timepoints. To identify which allelic states were likely ancestral, we extracted the reconstructed sequence of the ancestral node that contains our target species based on an alignment of 363 bird assemblies from Feng et al. (2020) and mapped it to our reference genome with the default parameters of BWA-MEM (Li and Durbin 2009). This node contains three sister species; *Myiagra hebetior* (estimated divergence time 12 MYA), *Paradisaea raggiana* and *Ifrita kowaldi* (estimated divergence time 25 MYA) (Jønsson et al. 2016; Kumar et al. 2022), and was assumed to represent the ancestral allele state. We randomly iterate over this dataset at two levels. First, to account for the variation due to different samples sizes between timepoints we randomly subsampled (with replacement) modern individuals to the same sample size as historical ones (*N* = 13). Second, to account for variation across the genome we randomly choose 1,000 filtered variants in each iteration. Sites and individuals were randomly sampled this way 100 times.

In each iteration, we tested (1) if there was a variant frequency difference between historical and modern samples, and (2) if historical and modern individuals had different numbers of deleterious alleles. (1) We estimated the relative frequency of putative deleterious variants between historical and modern time points per category using the R_xy_ approach described by Xue et al. (2015) following Dussex et al. (2021). Briefly, we estimated the per-site derived allele frequencies per timepoints (sFreq_hist_ and sFreq_mod_) and calculated the per-category frequency as cFreq_hist_ = ∑ sFreq_hist_(1−sFreq_mod_) and vice versa. We then estimated R_xy_ = cFreq_hist_/cFreq_mod_, where a value of 1 corresponds to no change in frequency, a value higher than 1 represents a deficit in the modern population, and a value lower than 1 represents an increase in the modern population. (2) We counted the total number of derived alleles per site per individual, and the count of those in homozygous state. The total count approximates the total genetic load in a sample, including mutations that do not express fitness effects (i.e. masked load) and those that fully or partially express their fitness effect (i.e. realized load). The homozygous counts approximate most of the realized load because these mutations fully express their deleterious effects. Partially recessive (heterozygous) deleterious mutations are also expected to partially express their deleterious effects, thus being part of the realized load (Bertorelle et al. 2022). However, dominance coefficients (*h*) of mildly and highly deleterious mutations are likely to be mostly recessive (Charlesworth and Willis 2009; supplementary fig. S19, Supplementary Material online) and thus are mostly part of the masked load (Bertorelle et al. 2022). Since the historical and modern samples have different sequence quality that impacts our ability to call SNPs, we corrected these derived allelic counts by dividing them by the total count of derived synonymous sites (i.e. low-impact variants), following Kuang et al. (2020). For each allele count comparison (across all iterations) we tested if the difference between historical and modern individuals was significant with the function t-test in R.

### Individual-Based Simulations

We performed individual-based forward simulations with SLiM v3.6 (Haller and Messer 2019) with a non-Wright-Fisher implementation. Absolute fitness (i.e. probability of survival) was regulated by genetic effects (see below) and the carrying capacity, which was determined with the reconstructed prebottleneck population size (see Results) and the known trajectory of the population decline and recovery (Bristol et al. 2013). We implemented three scenarios with different ancestral population sizes starting from the estimated ancestral population size, and population sizes that were 5 and 10 times larger (i.e. 1X, 5X, or 10X). We ran a burn-in for a number of generations that was five times the population size to obtain an ancestral population in mutation-selection-drift equilibrium. We ran 100 replicates per scenario.

To confirm that our model successfully replicated the overall biology of the Seychelles paradise flycatcher, we parameterized the model with known distributions for age-based mortality probability and litter size (Currie et al. 2005). We then analyzed the resulting full genealogy (with Tree sequence recording; Haller et al. 2019) to estimate the emerging generation time in the simulation, which matched the known generation time of ∼2 yr in this species (Bristol 2014, unpublished data).

Genetics parameters: we simulated 10,000 genes of 1 Kb each distributed across 28 autosomal chromosomes, typical of a passerine genome. We used a recombination rate of 1e-4 per base position, per generation, with no recombination within genes. We use a relatively large mutation rate of 1e-7 per bp to compensate for the small, simulated genome size and ensure the accumulation of genetic load in the ancestral populations.

To investigate the effect of the genetic load, we simulated deleterious mutations. We first investigated the relationship between selection (*s*) and dominance (*h*) coefficients in the distribution of fitness effects (DFE). For this, we conducted simulations with unconstrained mutations (supplementary fig. S19, Supplementary Material online; DFE0 in supplementary fig. S20, Supplementary Material online). Specifically, we drew values of *s* and *h* from uniform distributions (−1 < *s* < 0 and 0.5 < *h* < 0), allowing any combination of *s* and *h* to occur. Natural selection acts on this variation and a gamma distribution of DFE with a negative relationship between h and s naturally emerges from the simulations (supplementary fig. S19, Supplementary Material online). We randomly sampled 10,000 mutations in each replicate from the resulting simulated DFE to parametrize our simulations. Deleterious mutations appeared at a ratio of 2.31:1 relative to neutral mutations as observed in human exons (Kim et al. 2017). This distribution is approximately consistent with the predicted DFE of deleterious variation in humans (Eyre-Walker and Keightley 2007) meta-analysis (Charlesworth and Willis 2009) and experimental approaches (Agrawal and Whitlock 2012). Furthermore, we tested alternative DFEs previously used elsewhere (Kardos et al. 2021; Kyriazis et al. 2021; Pérez-Pereira et al. 2021; DFE1-DFE4 in supplementary fig. S20, Supplementary Material online) to compare the resulting trajectories of genetic load and probability of extinction over time (supplementary figs. S20 and S21, Supplementary Material online)

To investigate the effect of adaptive potential, we simulated the additive effect of genotype values (*z*) on a polygenic trait tracking an environmental optimum (*opt*). Genotype values (*z*) were drawn from a uniform distribution and with a fixed additive effect (*h* = 0.5). The effect of homozygous loci was estimated as Σz, the effect of the heterozygous loci as Σzh, and the phenotype (*P*) of an individual was the sum of the homozygous and heterozygous effects. Following Falconer and Mackay (1996), we calculated the fitness effect from the deviation of the phenotype to the environmental optimum as $w=(P-opt{)}^{2}$ and the additive genetic variation as $V_{A}=\Sigma 2p_{i}q_{i}z_{i}^{2}$. We performed an extensive parameter space exploration to test the effect of (i) the range from which genotype values (z) were drawn for the polygenic trait, and (ii) the relative proportion of mutation contributing to the adaptive trait relative to those contributing to the genetic load (supplementary figs. S22 to S24, Supplementary Material online). In the main text and Fig. 4, we present the results for simulations that take their genotype values (z) from uniform distribution ranging between −0.25 to 0.25 and with a proportion of 0.2 of mutation contributing to the adaptive trait relative to those contributing to the genetic load.

## Supplementary Material

msad256_Supplementary_Data

## Data Availability

The reference genome can be found at https://b10k.scifeon.cloud/#/b10k/Sample/S15237. The raw sequencing reads have been deposited in the Sequence Read Archive under the accession number PRJNA922178. Scripts can be found at https://github.com/hmoral/SPF
