# Supplementary material for: Genetic Load and Adaptive Potential of a Recovered Avian Species that Narrowly Avoided Extinction
Source: Mol Biol Evol. 2023 Nov 23;40(12):msad256. doi: 10.1093/molbev/msad256 (PMC10701096; doi:10.1093/molbev/msad256)
Supplement: msad256_Supplementary_Data [file msad256_supplementary_data.docx]

***Supplementary material for:***

**Genetic load and adaptive potential of a recovered avian species that narrowly avoided extinction**

Georgette Femerling^1,2,10^, Cock van Oosterhout^3^, Shaohong Feng^4,5,6^, Rachel M. Bristol^7,8^, Guojie Zhang^4,5,6^, Jim Groombridge^8^, M. Thomas P. Gilbert^1,9^ and Hernán E. Morales^1*^

^1^Globe Institute, Faculty of Health and Medical Sciences, University of Copenhagen, Copenhagen, Denmark

^2^ Centro de Ciencias Genómicas, Universidad Nacional Autónoma de México, Cuernavaca, México

^3^School of Environmental Sciences, University of East Anglia, Norwich, UK

^4^Center for Evolutionary & Organismal Biology, Zhejiang University School of Medicine, Hangzhou, 310058, China

^5^Liangzhu Laboratory, Zhejiang University Medical Center, 1369 West Wenyi Road, Hangzhou 311121, China

^6^Innovation Center of Yangtze River Delta, Zhejiang University, Jiashan 314102, China

^7^La Batie, Beau Vallon, Mahe, Seychelles

^8^Durrell Institute of Conservation and Ecology, School of Anthropology and Conservation, Division of Human and Social Sciences, University of Kent, Canterbury, Kent, CT2 7NR, United Kingdom.

^9^University Museum, NTNU, Trondheim, Norway

^10^Department of Human Genetics, McGill University, Montreal, Quebec, Canada

* Corresponding author - Hernán E. Morales (hernanm [at] sund.ku.dk)

**Table of contents**

[Table S1 Sample metadata 3](#_Toc149213371)

[Table S2 Diversity loss in bird species. 4](#_Toc149213372)

[Figure S1 Principal component analysis of historical and modern samples. 5](#_Toc149213373)

[Figure S2 Admixture analysis. 6](#_Toc149213374)

[Figure S3 Genetic diversity for 12 avian species in comparison to the historical and modern estimates for the Seychelles paradise flycatcher 6](#_Toc149213375)

[Figure S4 Depth of coverage tests on the principal component analysis. 7](#_Toc149213376)

[Figure S5 Depth of coverage tests on global heterozygosity and nucleotide diversity values. 8](#_Toc149213377)

[Figure S6 Depth of coverage tests on distribution of Runs of Homozygosity. 8](#_Toc149213378)

[Figure S7. Depth of coverage tests on the demographic reconstruction of population decline. 9](#_Toc149213379)

[Figure S8 Nucleotide diversity at ultra-conserved elements. 9](#_Toc149213380)

[Figure S9 No evidence of biases introduced by reference genome mapping. 10](#_Toc149213381)

[Figure S10 Genetic diversity loss across the genome. 11](#_Toc149213382)

[Figure S11 Genetic diversity loss across the genome vs. mapping quality. 11](#_Toc149213383)

[Figure S12 Genetic diversity loss across the genome vs. depth of coverage. 12](#_Toc149213384)

[Figure S13 Genetic diversity loss across the genome vs. DNA damage. 13](#_Toc149213385)

[Figure S14 Nucleotide diversity across the genome for historical and modern populations. 14](#_Toc149213386)

[Figure S15 Expected coalescence time for different Runs of Homozygosity (ROH) lengths. 15](#_Toc149213387)

[Figure S16. Fragment misincorporation plots for historical samples. 19](#_Toc149213388)

[Figure S17. Distribution of percentage of duplicated reads. 19](#_Toc149213389)

[Figure S18 Genetic relatedness in the modern samples. 20](#_Toc149213390)

[Figure S19 Distribution of fitness effects (DFE) simulations. 21](#_Toc149213391)

[Figure S20 Alternative distributions of fitness effects (DFE). 22](#_Toc149213392)

[Figure S21 Effect of alternative distributions of fitness effects (DFE) on the dynamics of the genetic load and extinction risk. 23](#_Toc149213393)

[Figure S22 Parameter test of forward simulations for the (Total) genetic load. 24](#_Toc149213394)

[Figure S23 Parameter test of forward simulations for the additive genetic variance in the quantitative trait (Va). 25](#_Toc149213395)

[Figure S24 Parameter test of forward simulations for the fitness effect conferred by the quantitative trait. 25](#_Toc149213396)

[References 25](#_Toc149213397)

## Table S1 Sample metadata

| **ID** | **Type** | **museum ID** | **Museum** | **Island** | **Year** | **Sex** | **Total Reads** | **Coverage** | **Read Len** | **Uniq map reads** | **Endo content** | **Soft-clip reads** | **Chimeric reads** |
| --- | --- | --- | --- | --- | --- | --- | --- | --- | --- | --- | --- | --- | --- |
| SPF1086 | Hist | 1887.12.30.1086 | Cambridge | La Digue | 1880 | M | 137318751 | 4.53 | 48 | 82682067 | 0.60 | 7874 | 0 |
| SPF261 | Hist | 1895.5.1.261 | Cambridge | Seychelles | 1880 | M | 157159682 | 5.17 | 48 | 93408489 | 0.59 | 8121 | 0 |
| SPF262 | Hist | 1895.5.1.262 | Cambridge | Seychelles | 1880 | M | 165408584 | 4.32 | 46 | 85932142 | 0.52 | 3195 | 0 |
| SPF2H | Hist | 1927.12.18.387 | NHM-UK | La Digue | 1888 | M | 173096670 | 6.09 | 53 | 106218309 | 0.61 | 39567 | 0 |
| SPF3H | Hist | 1881.11.14.1 | NHM-UK | Seychelles | 1880 | M | 152921326 | 4.33 | 49 | 81950883 | 0.54 | 6626 | 0 |
| SPF4I | Hist | 1927.12.18.390 | NHM-UK | La Digue | 1888 | F | 114759549 | 3.59 | 51 | 65038259 | 0.57 | 21893 | 0 |
| SPF4J | Hist | 1927.12.18.389 | NHM-UK | Praslin | 1888 | F | 106872254 | 3.4 | 51 | 61713152 | 0.58 | 13082 | 0 |
| SPF4K | Hist | 1927.12.18.386 | NHM-UK | La Digue | 1888 | M | 130099801 | 4.27 | 50 | 75750381 | 0.58 | 5374 | 0 |
| SPF5J | Hist | 1895.5.1.263 | NHM-UK | Seychelles | 1880 | F | 189425205 | 5.94 | 48 | 108193562 | 0.57 | 9251 | 0 |
| SPF5K | Hist | 1927.12.18.388 | NHM-UK | Praslin | 1888 | M | 162549025 | 6.18 | 56 | 98250630 | 0.60 | 66494 | 0 |
| SPF6D | Hist | 1881.11.14.2 | NHM-UK | Seychelles | 1880 | M | 136416796 | 4.9 | 51 | 86159116 | 0.63 | 11039 | 0 |
| SPF6F | Hist | 1881.11.14.3 | NHM-UK | Seychelles | 1880 | F | 161487071 | 5.15 | 51 | 89973978 | 0.56 | 22326 | 0 |
| SPF9 | Hist | 1988.21.9 | NHM-UK | Praslin | 1877 | M | 94230242 | 2.68 | 48 | 45962073 | 0.49 | 13427 | 0 |
| SPF02 | Mod | NA | NA | La Digue | 2007 | M | 61082413 | 9.37 | 171 | 59675013 | 0.98 | 2137234 | 337050 |
| SPF03 | Mod | NA | NA | La Digue | 2007 | M | 61466147 | 9.4 | 170 | 60050716 | 0.98 | 2112641 | 345431 |
| SPF04 | Mod | NA | NA | La Digue | 2007 | F | 60826617 | 9.36 | 171 | 59433082 | 0.98 | 2060139 | 323045 |
| SPF05 | Mod | NA | NA | La Digue | 2007 | M | 61168467 | 9.38 | 171 | 59756550 | 0.98 | 2117661 | 340986 |
| SPF06 | Mod | NA | NA | La Digue | 2007 | Unk | 59732978 | 9.19 | 172 | 58134693 | 0.97 | 3373123 | 320737 |
| SPF07 | Mod | NA | NA | La Digue | 2008 | Unk | 62018705 | 9.49 | 171 | 60328829 | 0.97 | 3175310 | 352950 |
| SPF08 | Mod | NA | NA | La Digue | 2008 | M | 57977143 | 8.97 | 173 | 56601669 | 0.98 | 3711159 | 291785 |
| SPF09 | Mod | NA | NA | La Digue | 2008 | Unk | 61566996 | 9.46 | 172 | 59788806 | 0.97 | 3353558 | 333061 |
| SPF10 | Mod | NA | NA | La Digue | 2008 | Unk | 61251614 | 9.36 | 171 | 59701392 | 0.97 | 3653747 | 332174 |
| SPF11 | Mod | NA | NA | La Digue | 2008 | M | 54359034 | 8.44 | 175 | 52588442 | 0.97 | 1578883 | 289160 |
| SPF12 | Mod | NA | NA | La Digue | 2008 | M | 62359375 | 9.55 | 172 | 60385746 | 0.97 | 1702602 | 340339 |
| SPF13 | Mod | NA | NA | La Digue | 2008 | F | 57069556 | 8.78 | 173 | 55174295 | 0.97 | 1904220 | 323704 |
| SPF14 | Mod | NA | NA | La Digue | 2008 | Unk | 58813711 | 9.22 | 177 | 56792584 | 0.97 | 1786421 | 292595 |
| SPF15 | Mod | NA | NA | La Digue | 2008 | Unk | 54319648 | 8.08 | 175 | 50260459 | 0.93 | 1787184 | 294236 |
| SPF16 | Mod | NA | NA | La Digue | 2008 | F | 60440888 | 9.27 | 176 | 57351550 | 0.95 | 1757734 | 323178 |
| SPF17 | Mod | NA | NA | La Digue | 2008 | M | 61092454 | 9.01 | 172 | 57207466 | 0.94 | 2206575 | 323293 |
| SPF18 | Mod | NA | NA | La Digue | 2008 | M | 61202354 | 9.12 | 173 | 57320772 | 0.94 | 1759823 | 332691 |
| SPF19 | Mod | NA | NA | La Digue | 2008 | M | 57420195 | 8.59 | 177 | 52916398 | 0.92 | 1810002 | 293949 |
| SPF20 | Mod | NA | NA | La Digue | 2008 | M | 63262323 | 9.72 | 172 | 61527191 | 0.97 | 1874952 | 338235 |

## Table S2 Diversity loss in bird species.

Loss of genomic diversity of different bird species when compared with historical values from museum-preserved samples. Two different metrics are reported: pairwise nucleotide diversity (Pi) and average heterozygosity (He). Year range and generation range refer to the range of time/generation that spans between historical and contemporary samples. For comparison the values we reported for the Seychelles paradise flycatcher were a 6.4-fold loss in He, 10-fold loss in Pi for a comparison that spans a range of 120-131 years and 60-65 generations (see main text). Methodological differences in how the metrics were calculated (e.g., filtering parameters) could introduce small biases, so while the estimates are not directly comparable, these differences are likely minimal, and the overall patterns should not be compromised

| Species | Generation time | Metric | Delta (fold loss) | Historical sample years | Year range | Generation range | Reference |
| --- | --- | --- | --- | --- | --- | --- | --- |
| Crested Ibis | 3 | Pi | 1.85 | 1841-1922 | 177-100 years | 56-32 | [Feng et al. (2019)](https://paperpile.com/c/8Rwn1d/8Al2) |
| Chatham Island black robin | 2 | Pi | 1.8 | 1871-1900 | 143-114 years | 72-57 | [von Seth et al. (2022)](https://paperpile.com/c/8Rwn1d/popcy) |
| South Island Saddleback | 8 | He | 4.16 | 1877–1898 | 128-107 years | 16 | [Taylor et al. (2007)](https://paperpile.com/c/8Rwn1d/pPfnM) |
| New Zealand Robin | 4 | He | No significant loss | 1873-1955 | 127-50 years | 31-12 | [Taylor et al. (2007)](https://paperpile.com/c/8Rwn1d/pPfnM) |
| Dutch black grouse | 3 | He | No significant loss | 1893-1953 | 115-55 years | 38-18 | [Segelbacher et al. (2014)](https://paperpile.com/c/8Rwn1d/O4ws) |
| Mangrove Finch (Isabella) | 1 | He | 1.32 | 1899 | 100-114 years | 100-114 | [Lawson et al. (2017)](https://paperpile.com/c/8Rwn1d/lznvx) |
| Greater Prarie Chicken | 2 | He | 1.26 | 1951 | 45 years | 22 | [Bellinger et al. (2003)](https://paperpile.com/c/8Rwn1d/iWXqL) |


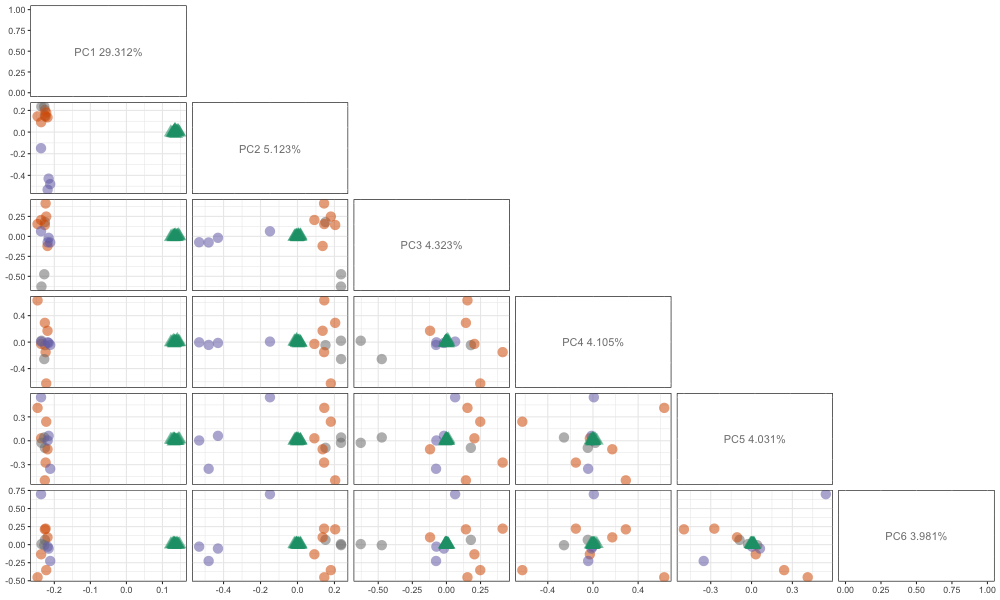


## Figure S1 Principal component analysis of historical and modern samples.

Historical: circles; (La Digue-orange and Praslin-purple) and Modern: triangles (La Digue-green). One of the challenges of working with old museum-preserved samples is that their associated metadata often lacks precise information about their provenance. In our case, 6 samples lacked information on which island the individuals were collected on. However, using the clear signal of population structure from the PCA we were able to assign those historical individuals to their respective islands.


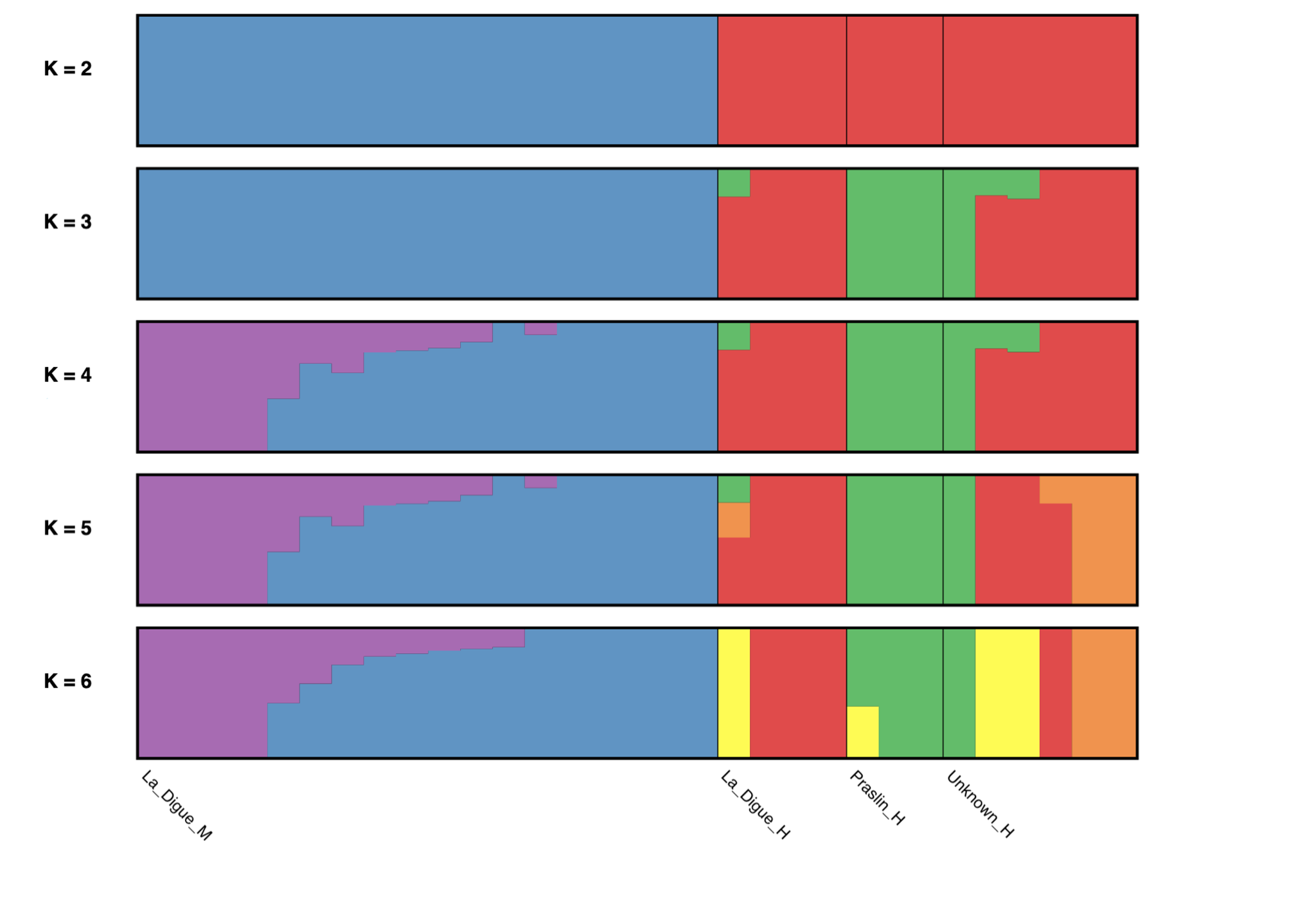


## Figure S2 Admixture analysis.

Visualization of admixture proportions of Modern (La_Digue_M) and Historical (La_Digue_H, Praslin_H, Unknown_H) samples of the best run per K, from K=2-6. Unknown_H groups historical samples with missing sampling location metadata. Colours represent ancestry components. Each bar represents a sample, and samples are sorted by the component with the highest proportion in the group in the highest K. Sample order is the same across all Ks.

**
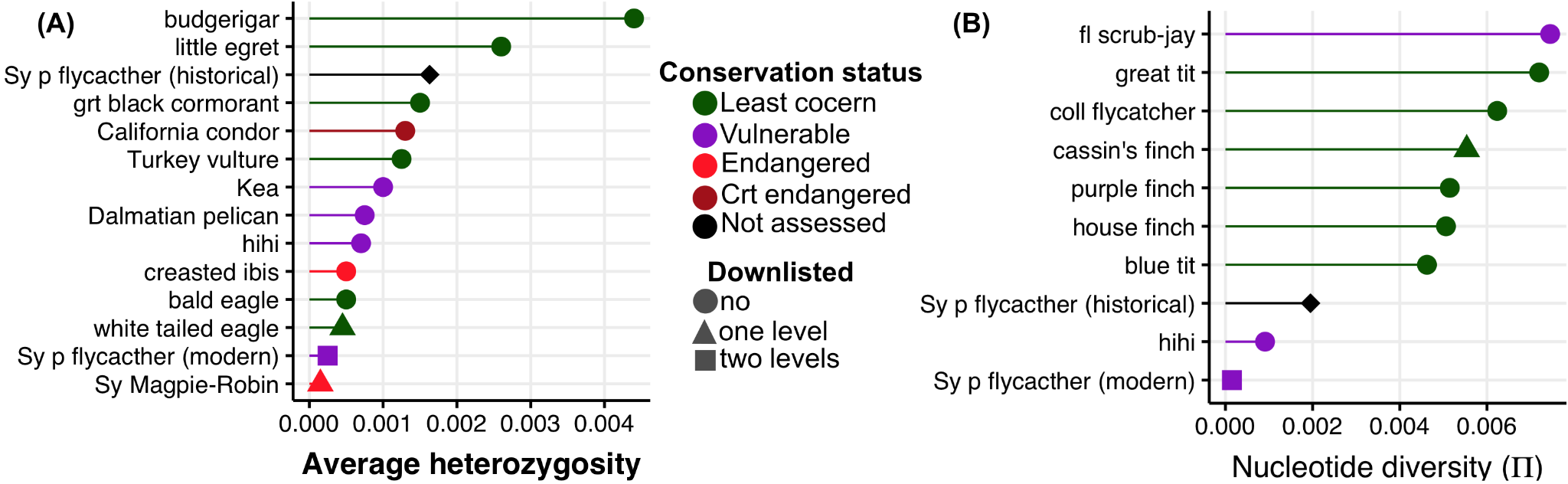

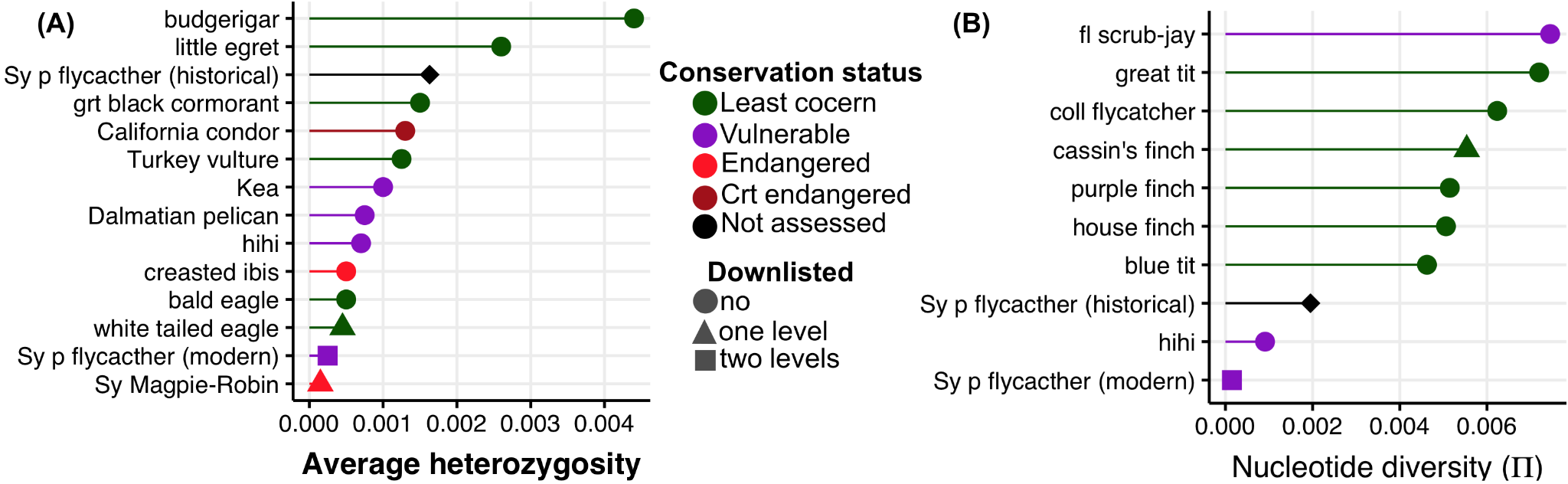
**

## Figure S3 Genetic diversity for 12 avian species in comparison to the historical and modern estimates for the Seychelles paradise flycatcher

Colours denote conservation status and the symbol denotes how many steps the species has been downlisted in the IUCN Red List. (A) Average genome-wide average heterozygosity. The figure was adapted from [(Cavill et al. 2022)](https://paperpile.com/c/8Rwn1d/5KAqL). Data for nine is from [(Li et al. 2014)](https://paperpile.com/c/8Rwn1d/hMofM), one from [(de Villemereuil et al. 2019)](https://paperpile.com/c/8Rwn1d/CF7rL) and one from [(Robinson et al. 2021)](https://paperpile.com/c/8Rwn1d/h8MxV). (B) Average genome-wide nucleotide diversity (π). The figure was adapted from [(de Villemereuil et al. 2019)](https://paperpile.com/c/8Rwn1d/CF7rL) with data from [(Chen et al. 2014; Shultz et al. 2016; Dutoit et al. 2017; Charles Perrier et al. 2018; C. Perrier et al. 2018)](https://paperpile.com/c/8Rwn1d/hWmvz+mWdT0+RECR5+uGdbr+07dj5). Methodological differences in how the metrics were calculated (e.g., filtering parameters) could introduce small biases, so while the estimates are not directly comparable, these differences are likely minimal, and the overall patterns should not be compromised.


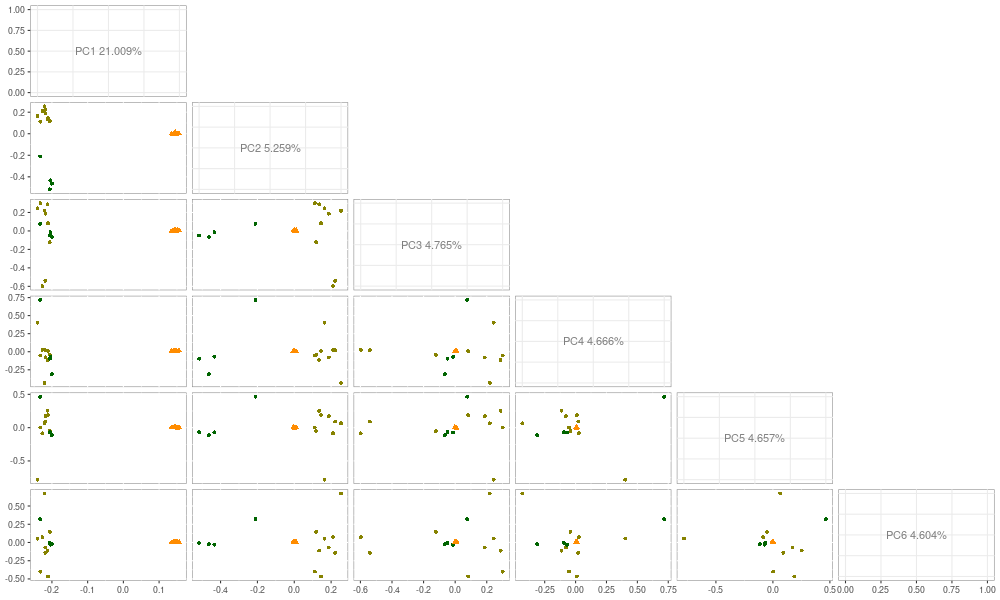


## Figure S4 Depth of coverage tests on the principal component analysis.

Principal component analysis using downsampled (4x) modern samples to match the average depth of coverage of historical samples. Results are similar across all PCs between the full coverage and down sampled runs.


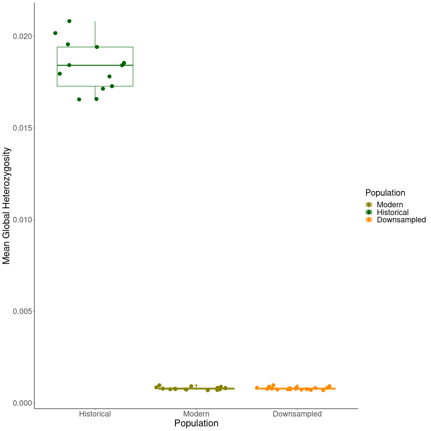

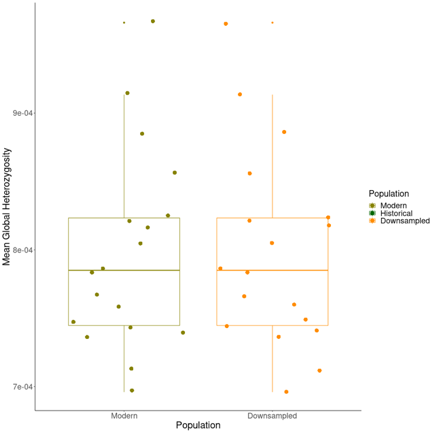

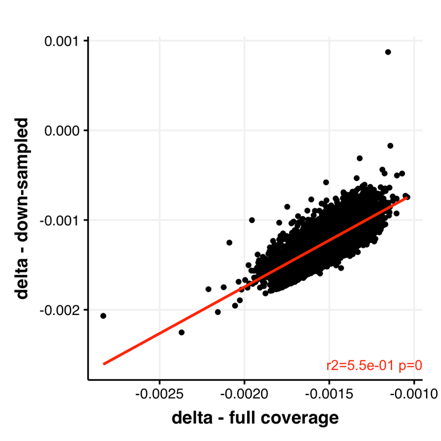


## Figure S5 Depth of coverage tests on global heterozygosity and nucleotide diversity values.

Left panel shows heterozygosity levels between the modern and historical samples including full coverage modern samples (~9X) and downsampled (4x) modern samples to match the average depth of coverage of historical samples. Middle panel shows a direct comparison between full-coverage and downsampled modern samples. Left panel shows a comparison between delta nucleotide diversity (π) estimated with full coverage modern samples (~9X) and with sub-sampled modern sampled to the depth of coverage of the historical samples (~4X).


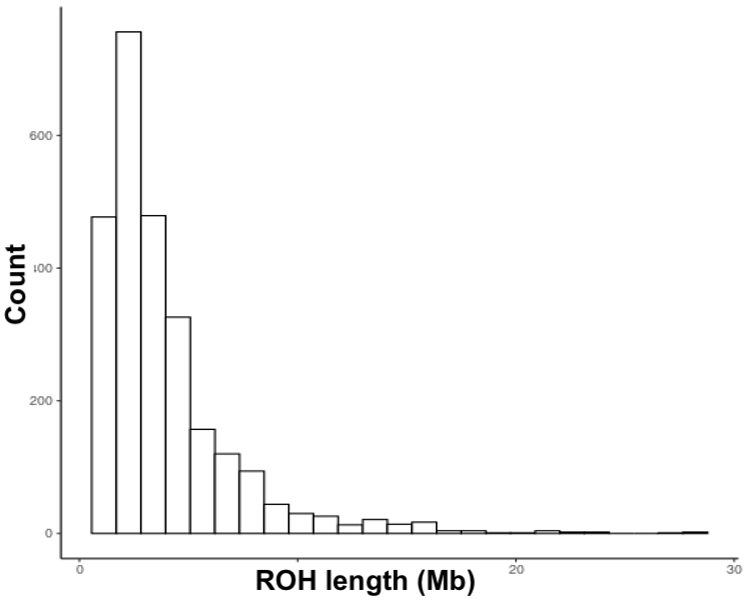


## Figure S6 Depth of coverage tests on distribution of Runs of Homozygosity.

Runs of homozygosity (ROH) length distribution across all modern individuals downsampled (4x) to match the average depth of coverage of historical samples. Distribution is similar to that of the full-coverage modern samples (Fig. 2 of the main text), with the most common category of ROH being between 1-2 MB of length.


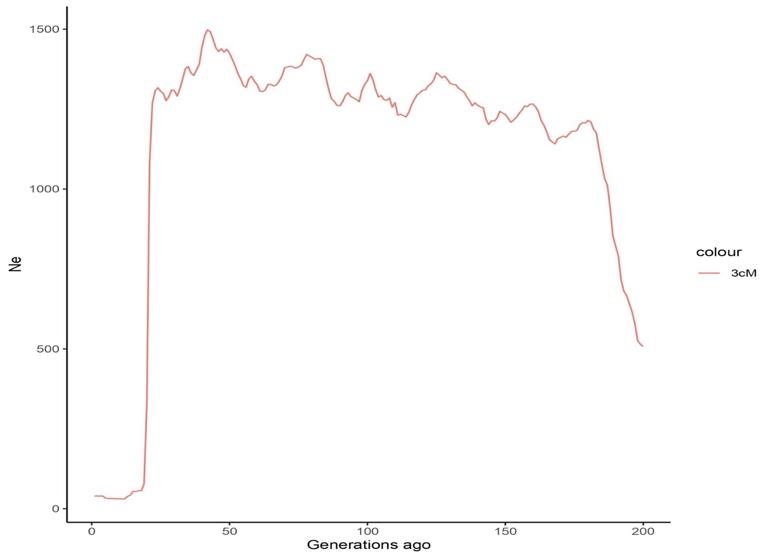


## Figure S7. Depth of coverage tests on the demographic reconstruction of population decline.

Reconstruction of the recent demography (last 200 generations) from Linkage Disequilibrium using GONE [(Santiago et al. 2020)](https://paperpile.com/c/nozmyi/tA5T) assuming a recombination rate of 3 cM/Mb. Modern samples were downsampled to 4x to match the average depth of coverage of historical samples. The result is very similar to that of the full-coverage modern samples (Fig. 2 of the main text).


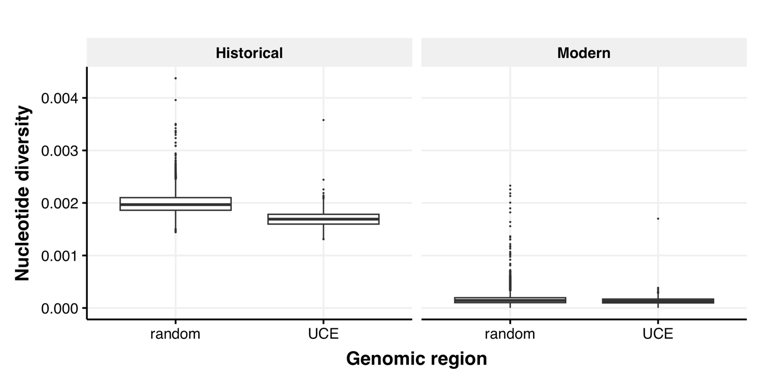


## Figure S8 Nucleotide diversity at ultra-conserved elements.

We downloaded the UCE sequences from Feng et al. (2020; 400-1500 bp regions). These UCE sequences were extracted for their Cactus alignment of 363 bird assemblies following the PHYLUCE pipeline (see Supplementary Information of Feng et al 2020). We blasted the UCE sequences against our genome, retaining >2,700 sequences that mapped uniquely (e value < 1e6). We estimated nucleotide diversity in 500bp windows (see Methods main text) and summarised their variation at overlapping UCE windows and at the same number of random (nonUCE) windows. While evidence of high nucleotide diversity in UCE regions might be of concern and indicative of DNA damage, UCE are expected to have a low rate of substitution and mutation at low frequency, but not necessarily low diversity. An analysis of 29,938 polymorphisms within 2189 UCEs in the human genome (Habic et al. 2019), showed that the polymorphism density within UCEs (one per 22.7 bp) was higher than the genomic average (one per 21.0 bp). However, the frequency of the derived allele was extremely low (less than 6% occur at a frequency of >1%), which suggests that UCE are kept under strong purifying selection.


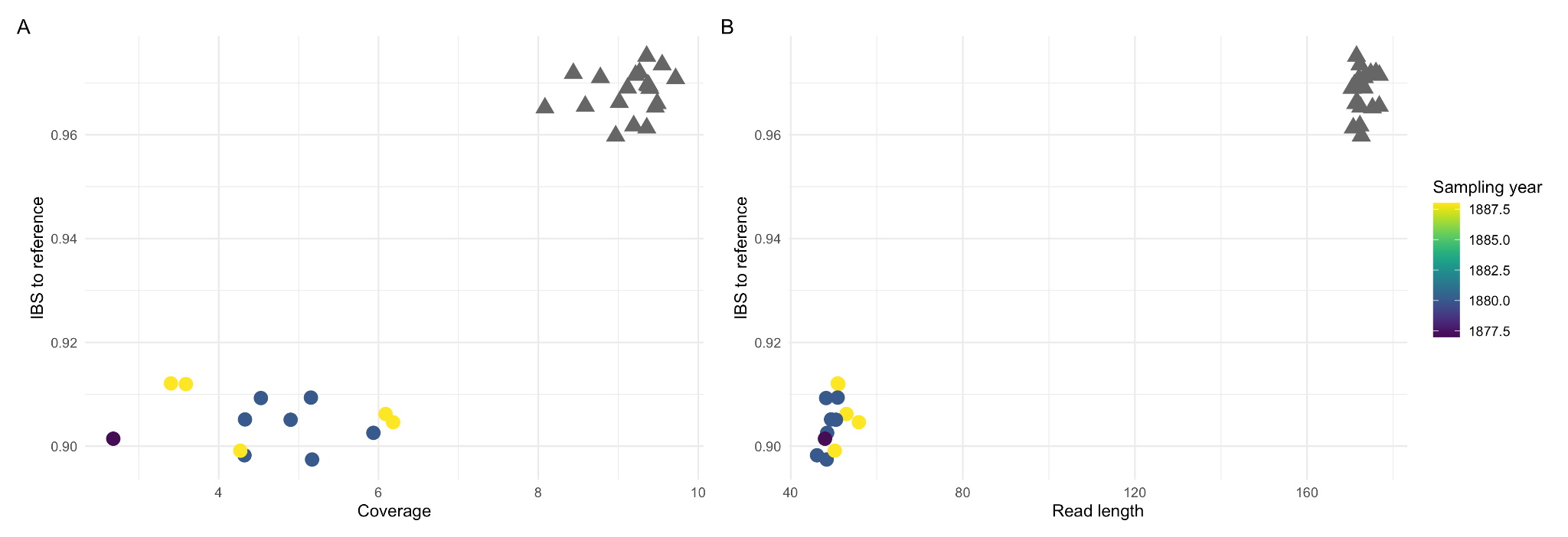


## Figure S9 No evidence of biases introduced by reference genome mapping.

The relatively short average read lengths common to ancient DNA (aDNA) caused by post-mortem DNA damage can cause sequence miss-alignments and generate an incorrect tendency to call SNPs that are biased towards the reference genome [(Liu et al. 2021; Gopalakrishnan et al. 2022)](https://paperpile.com/c/8Rwn1d/TBj1K+mRnG8). Thus, if this bias is present, the expectation is for historical samples to have allele calls that are (artificially) more like the reference genome, i.e., a smaller identity-by-state (IBS) distance to the reference genome. Moreover, low coverage can also create artefacts on the ability to correctly call alleles, with lower coverage disproportionately being called with the reference allele. On the other hand, if DNA damage was not pervasive enough to create a bias, we expect the historical samples to have a higher IBS distance to the modern reference genome, due to drift and loss of diversity. Here we confirmed that our historical dataset does not suffer from mapping reference biases as older historical samples (circles) have a higher IBS distance to the reference genome than the modern samples (triangles) and their IBS values are not explained by either (A) coverage or (B) read length. We also confirm the relationship is the other way around, with historical samples having a smaller identity-by-state (IBS) distance to the reference genome, as expected on well behaving historical samples. To obtain IBS, we randomly sampled one base from the alignments with ANGSD v0.921[(Korneliussen et al. 2014)](https://paperpile.com/c/8Rwn1d/ZLTa9) and counted the instances that the allele was the same as the reference genome allele.


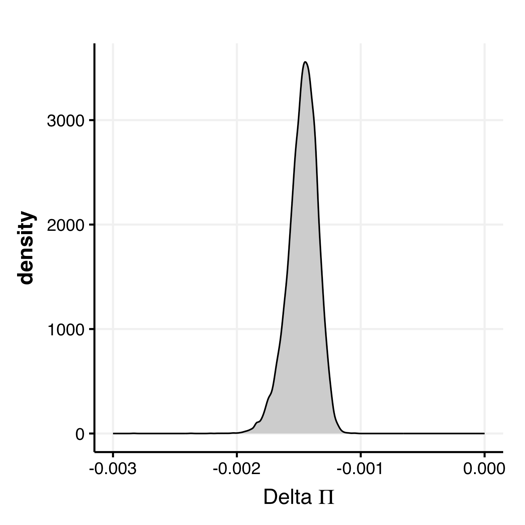


## Figure S10 Genetic diversity loss across the genome.

Density distribution of delta (i.e., modern minus historical) nucleotide diversity (π) across the genome for sliding windows of 50 Kb


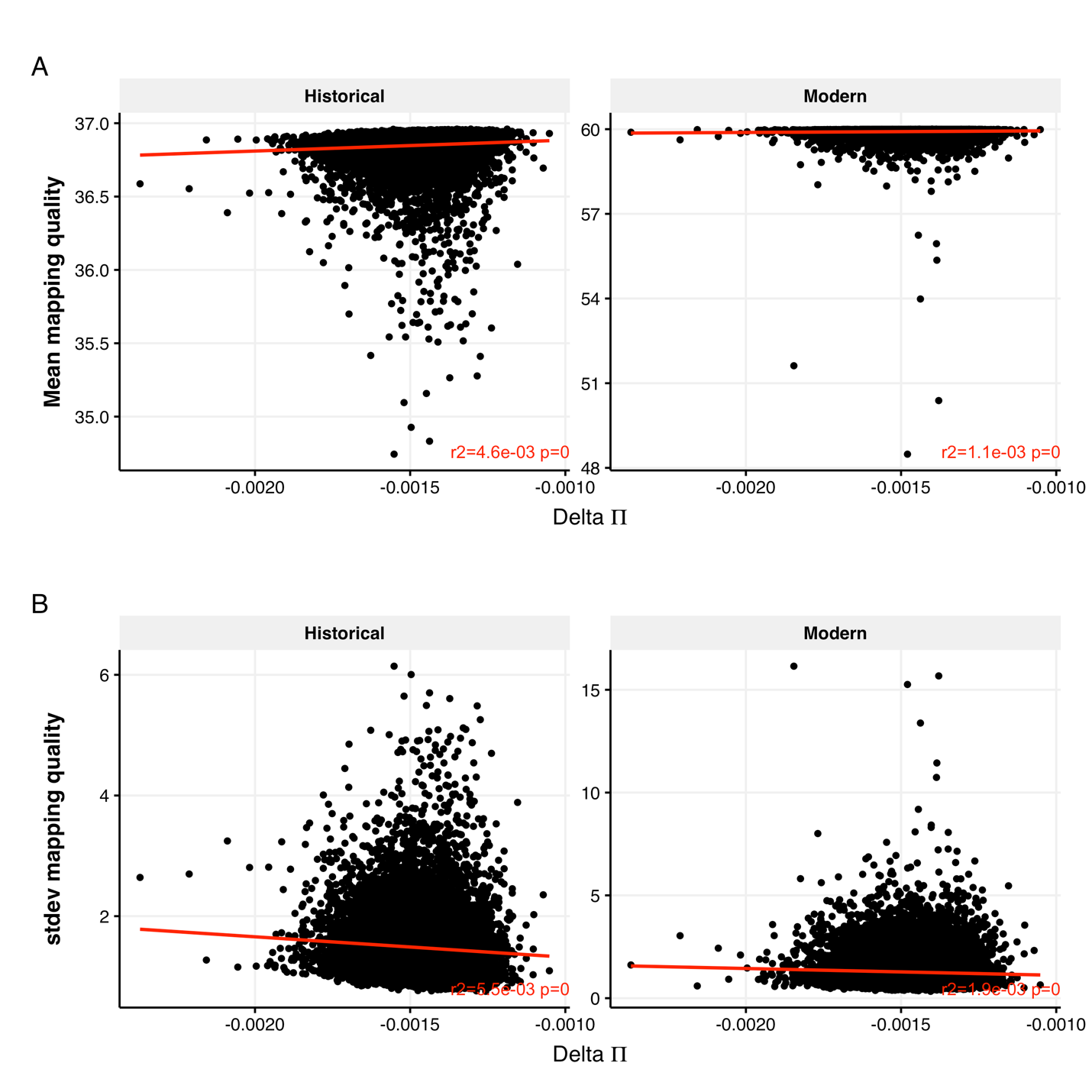


## Figure S11 Genetic diversity loss across the genome vs. mapping quality.

Comparison between delta nucleotide diversity (π) and mapping quality across the genome for sliding windows of 50 Kb (A) Mean mapping quality per window. (B) Standard deviation of mapping quality values per window. Panels show correlation for historical and modern samples. Fit linear model, r^2^ and p-value are shown in red.


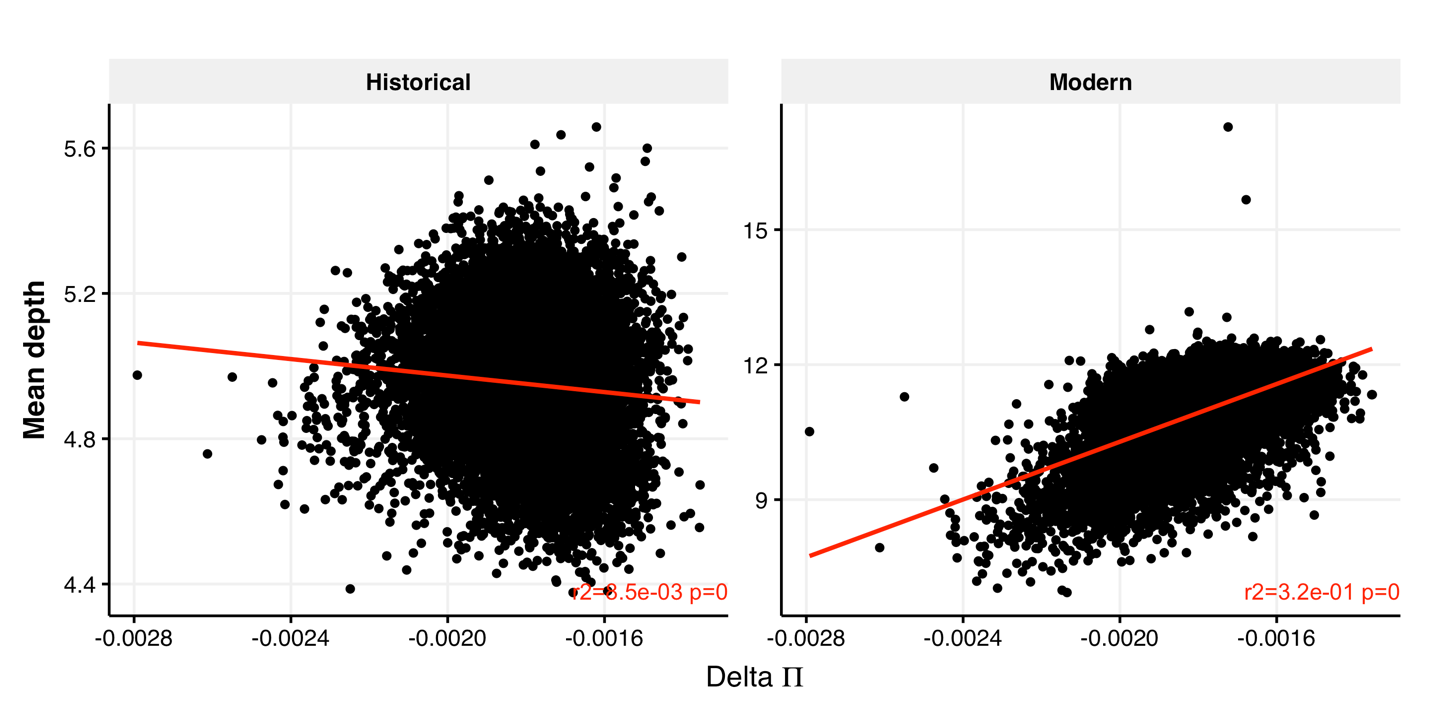


## Figure S12 Genetic diversity loss across the genome vs. depth of coverage.

Comparison between delta nucleotide diversity (π) and mean depth of coverage across the genome for sliding windows of 50 Kb. Panels show correlations for historical and modern samples. Fit linear model, r^2^ and p-value are shown in red.


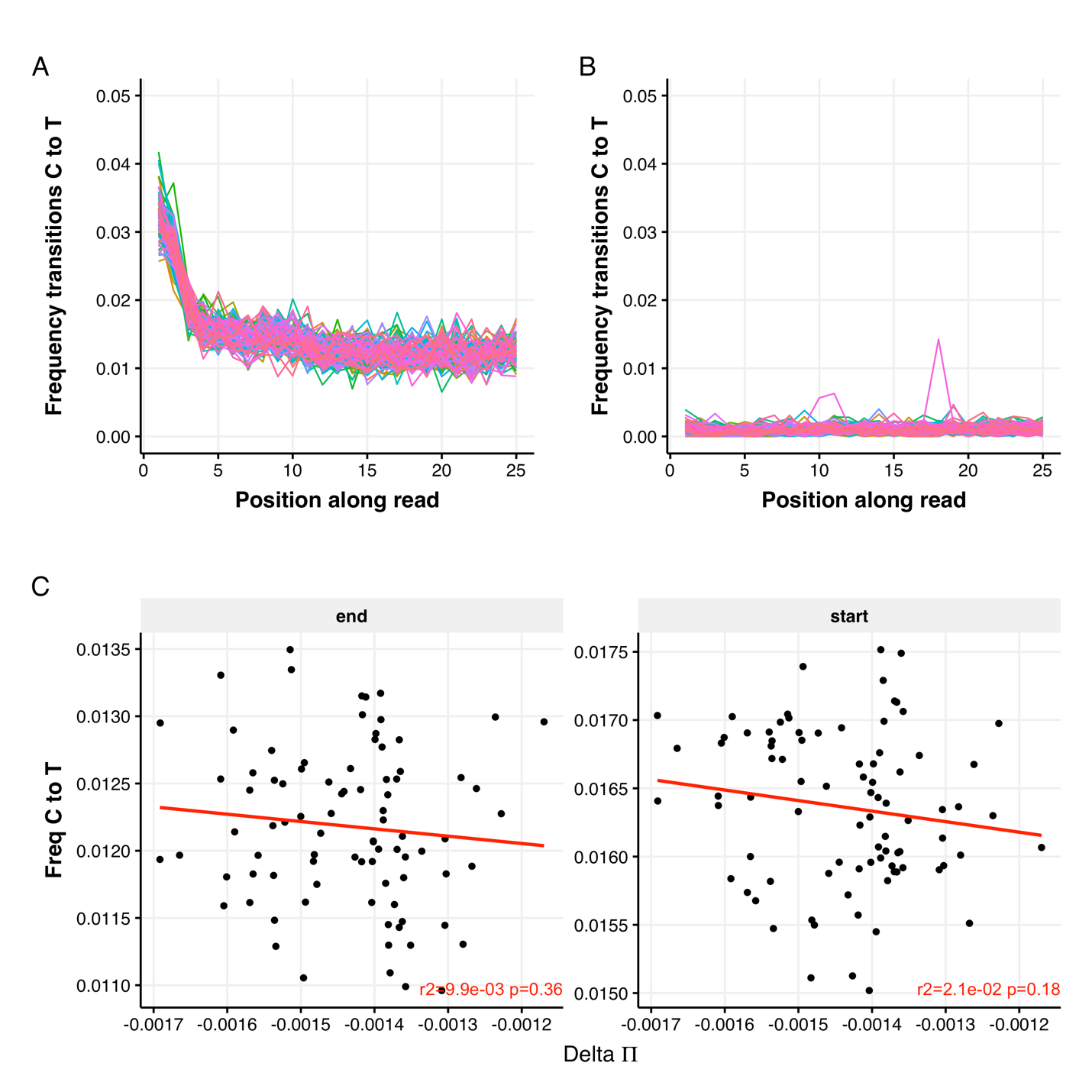


## Figure S13 Genetic diversity loss across the genome vs. DNA damage.

Comparison between delta nucleotide diversity (π) and DNA damage for 100 windows of 50 Kb randomly sampled across the genome. (A) Historical samples show a typical pattern of DNA damage as a higher frequency of C to T transitions, particularly at the start of the sequencing read. (B) Modern samples do not show the same pattern of DNA damage. (C) We averaged the values for the start (first 10 bp) and the end (last 10 bp) of the sequencing read and compare it against delta π. Fit linear model, r^2^ and p-value are shown in red.


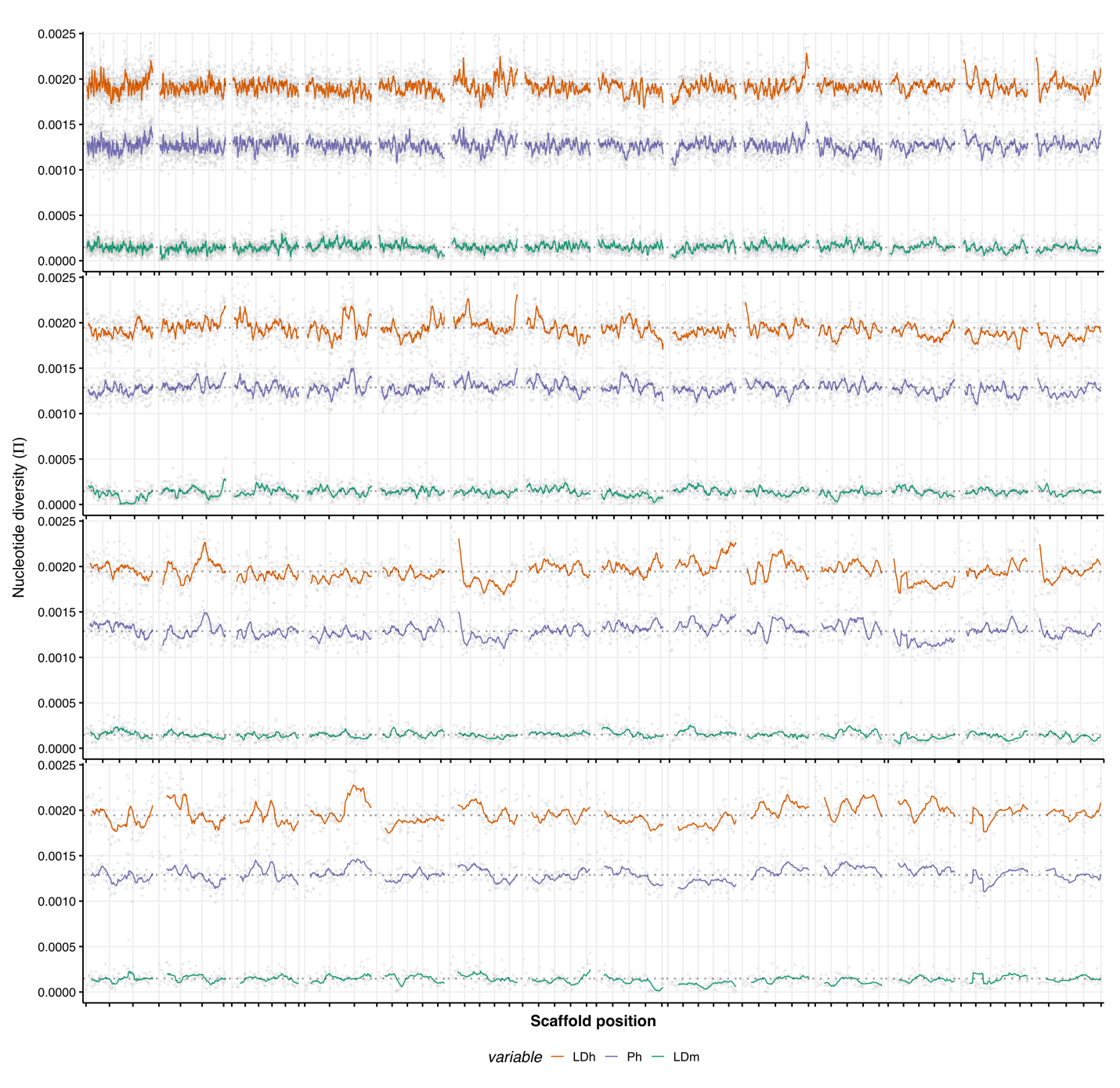


## Figure S14 Nucleotide diversity across the genome for historical and modern populations.

The panels represent continuous runs of scaffolds ordered from long to short. We estimated genome-wide nucleotide diversity (π) from the population-level folded Site Frequency in ANGSD [(Korneliussen et al. 2014)](https://paperpile.com/c/8Rwn1d/ZLTa9) following [Korneliussen et al. (2013)](https://paperpile.com/c/8Rwn1d/lk49y) in non-overlapping sliding windows of 50 Kb. Here we plot the 59 autosomal scaffolds longer than 4Mb, which represent ~60% of the total reference genome length


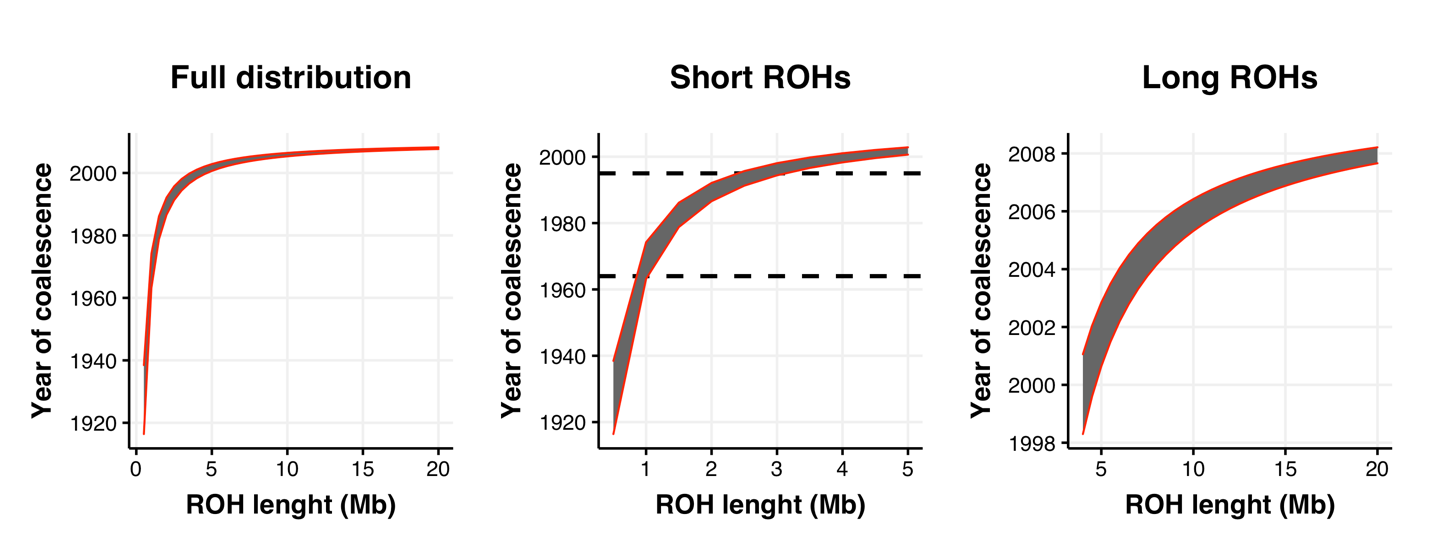


## Figure S15 Expected coalescence time for different Runs of Homozygosity (ROH) lengths.

ROHs are formed when very closely related individuals mate (i.e., inbreeding) and inherit identical segments of DNA to their offspring. As time passes, and if the rate of inbreeding reduces, ROHs are “broken down” by recombination and become shorter. Thus, by estimating the length of a ROH and assuming a constant recombination rate we can calculate their coalescence time, i.e. the time at which the ROH was likely formed. We used the formula *L = 100/2t cM* [(Thompson 2013)](https://paperpile.com/c/8Rwn1d/npGd9) where *L* is the length of the ROH, *cM* is the recombination rate and *t* is the unknown time of coalescence in generations. We used a range of recombination rate values 1.71- 3.56 cM/Mb from different bird species (collared flycatcher: [(Kawakami et al. 2017)](https://paperpile.com/c/8Rwn1d/krHXE); zebra finch: [(Backström et al. 2010)](https://paperpile.com/c/8Rwn1d/NfThE) and Helmeted honeyeater: [(Robledo-Ruiz et al. 2022)](https://paperpile.com/c/8Rwn1d/YkuKu)). We converted generation-ago estimates to years assuming an average generation time of 2 for the Seychelles paradise flycatcher (R. Bristol unpublished). (A) Coalesce times for ROHs length 0.5-20 Mb (B) Coalesce times for short ROH <5 Mb. The approximate time of the bottleneck is indicated in a dashed line. ROHs that formed during the bottleneck should be 1-2 Mb long. (B) Coalesce times for long ROHs >5 Mb that should be formed due to recent inbreeding. In all panels, the shading indicates the 95% confidence intervals obtained using the 1.71- 3.56 cM/Mb range of recombination rates.

SPF2H


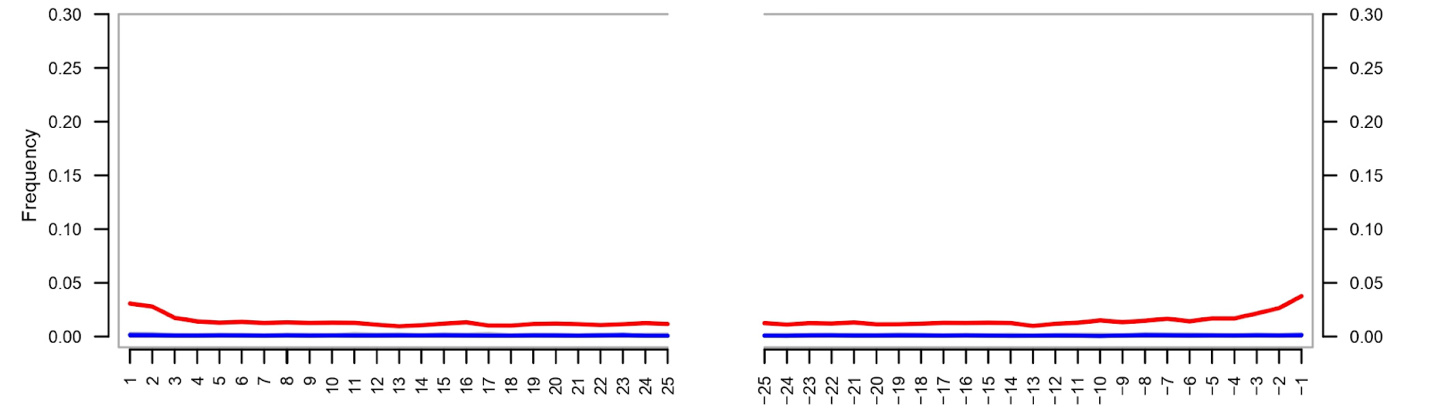


SPF4I


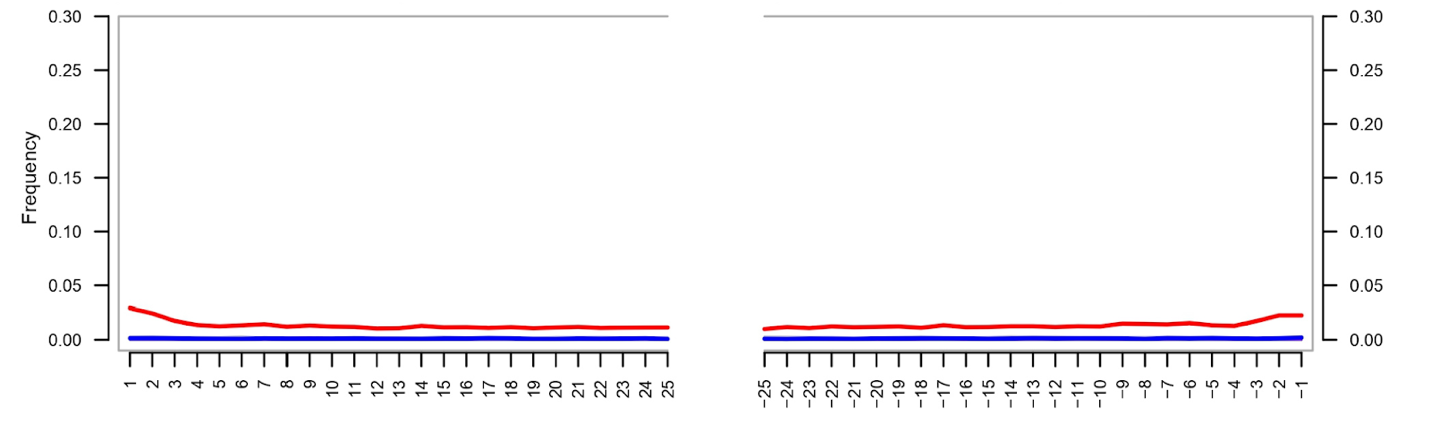


SPF3H


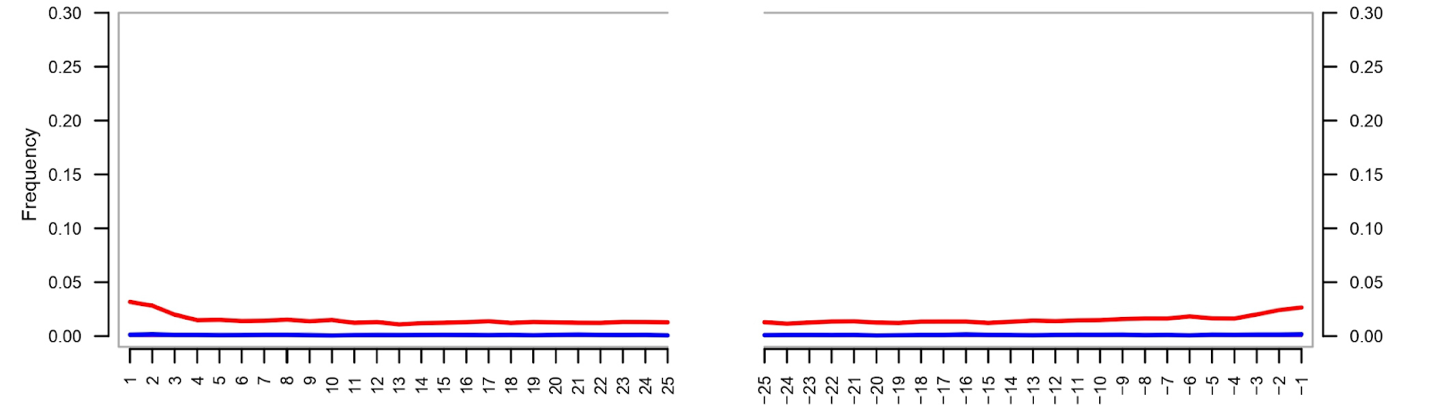


SPF4J


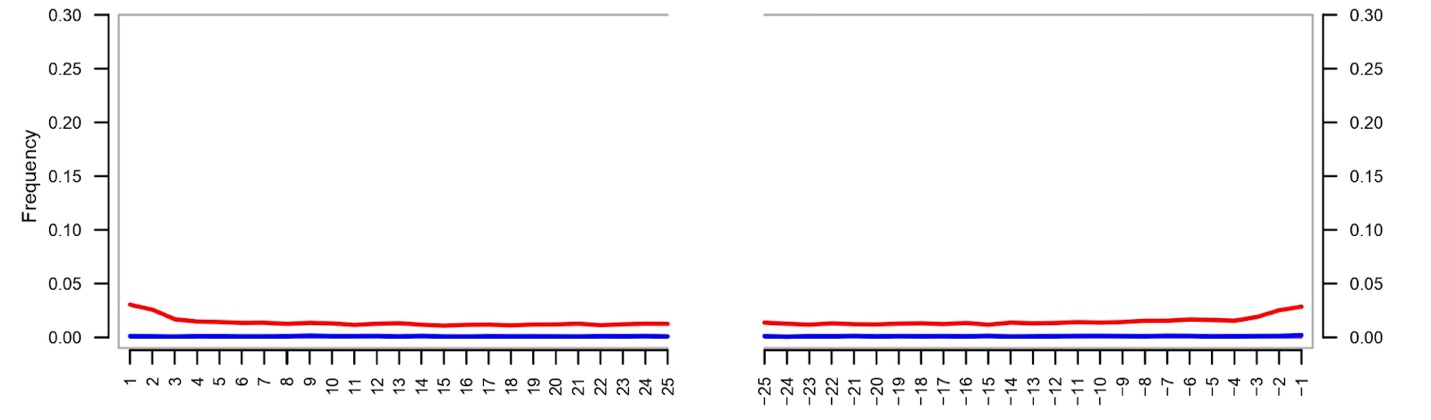


SPF4K


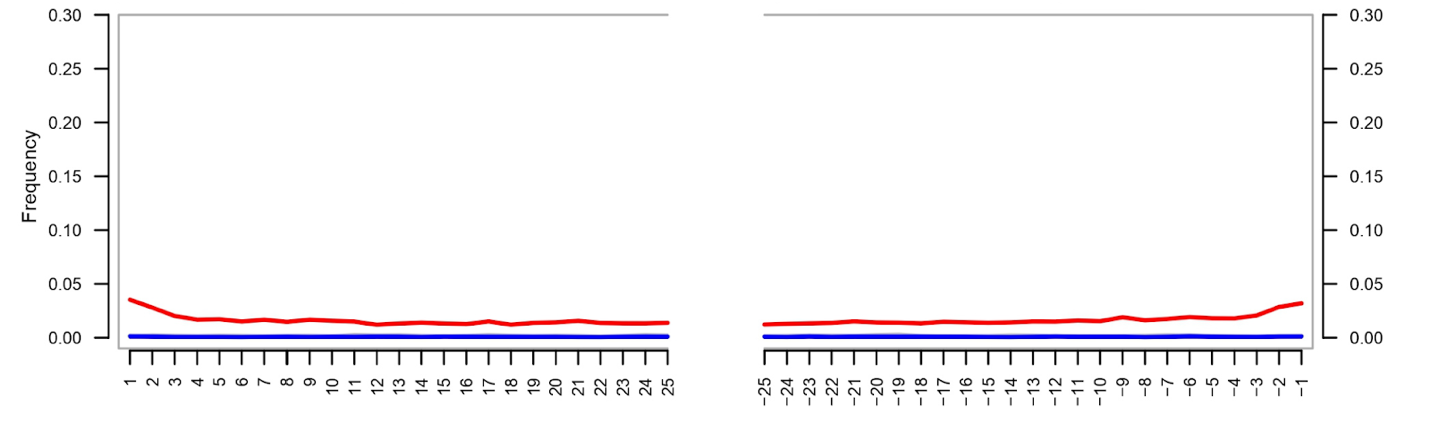


SPF5K


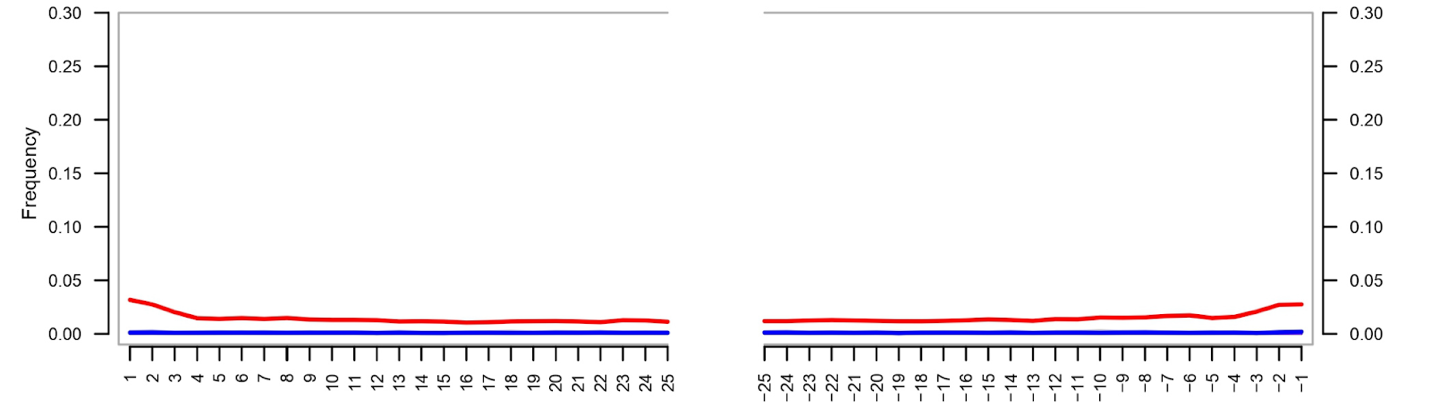


SPF5J


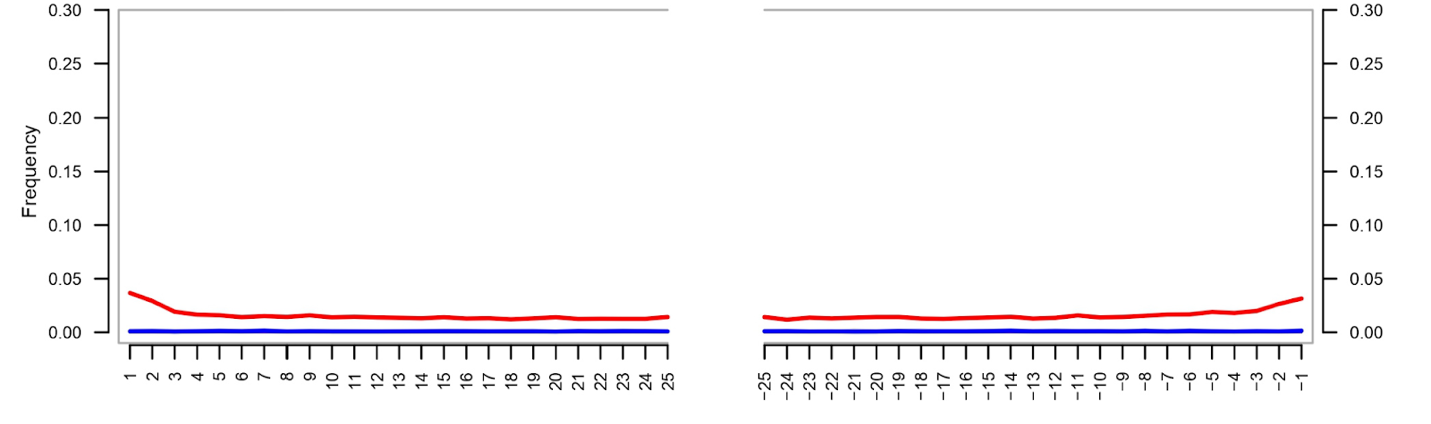


SPF6D


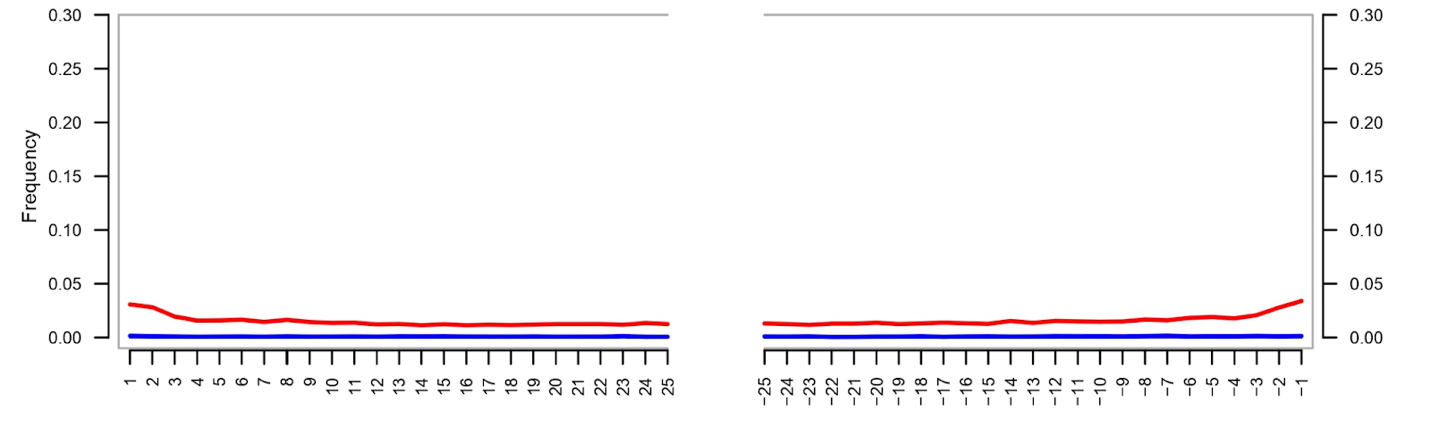


SPF6F


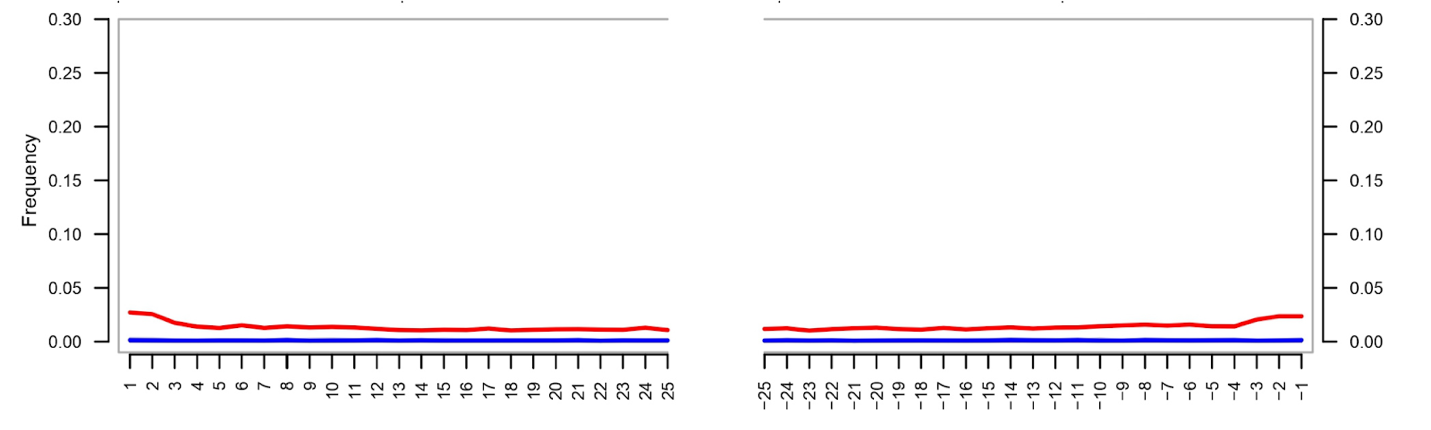


SPF9


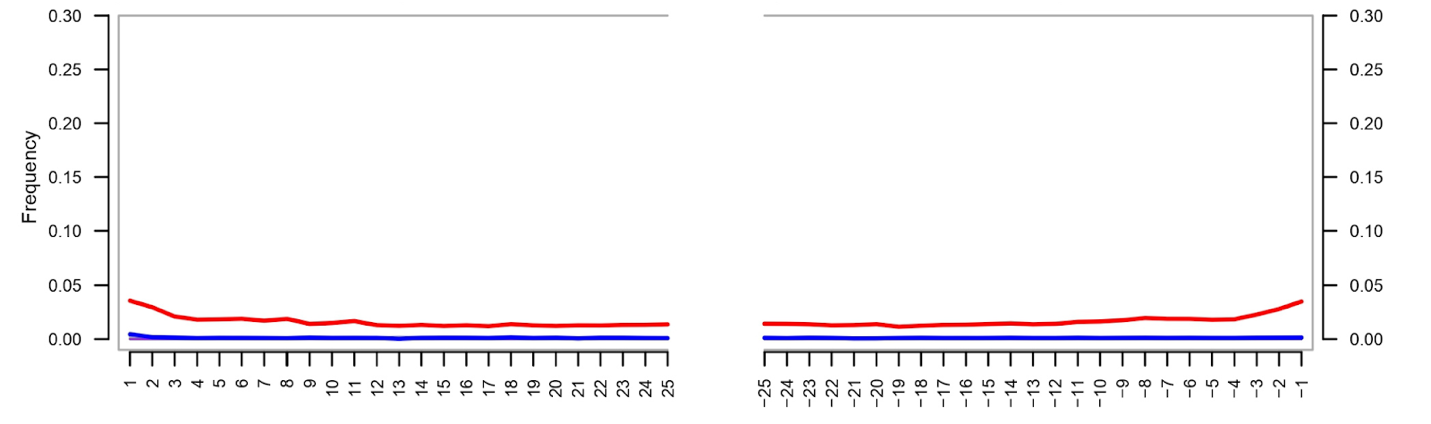


SPF261


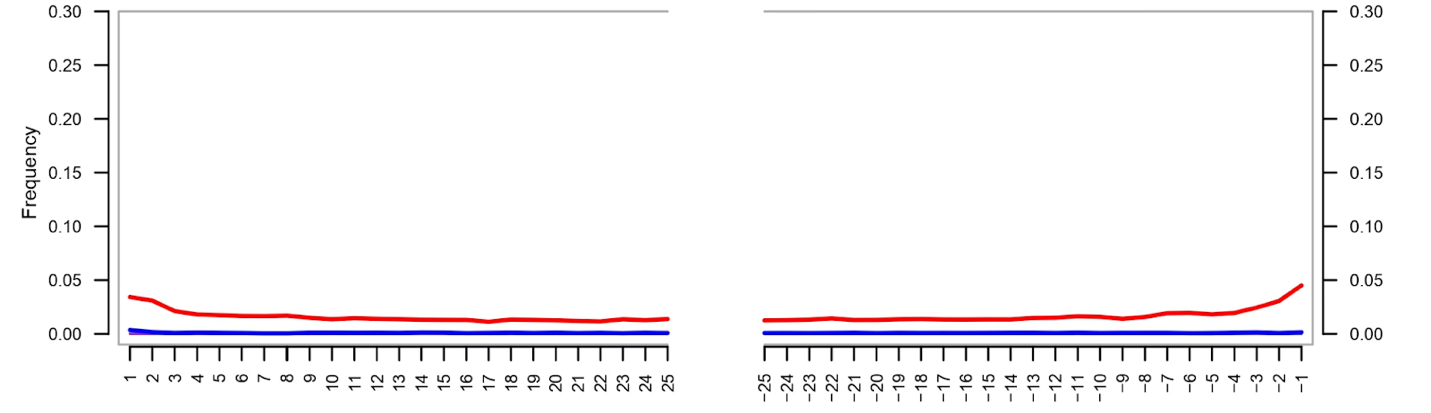


SPF262


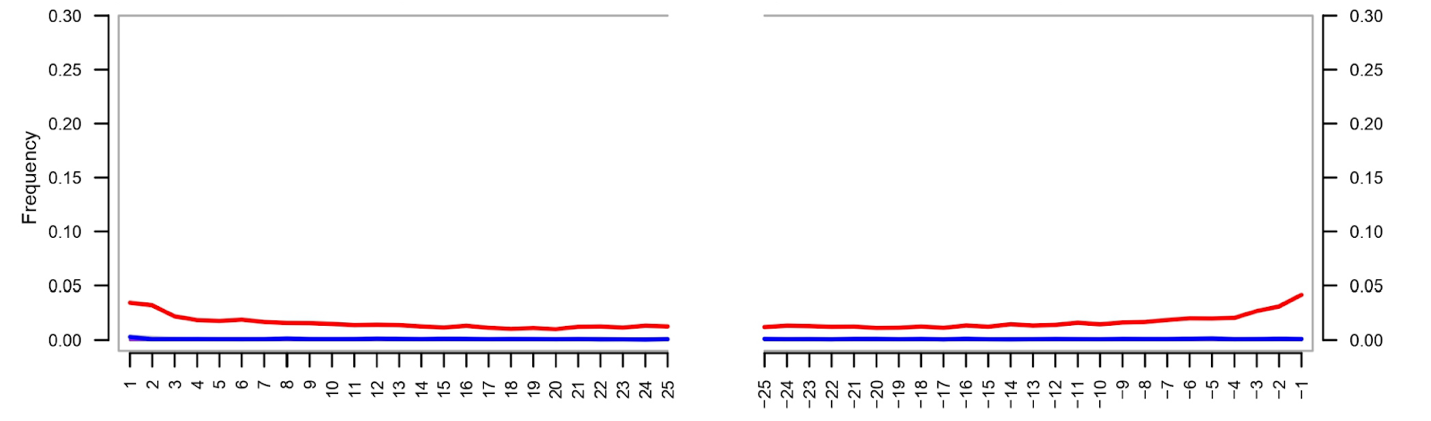


SPF1086


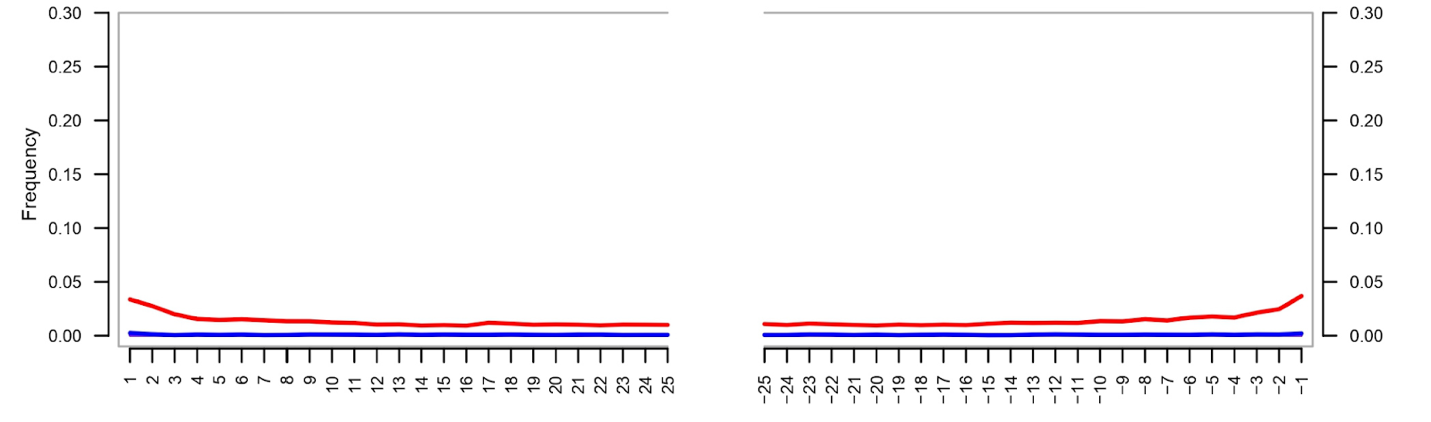


## Figure S16. Fragment misincorporation plots for historical samples.

Each panel corresponds to one historical sample and shows the DNA damage. The red line indicates transitions from C to T, and the blue line indicates the transitions from G to A. Y-axis shows the proportion of sites with that nucleotide change and x-axis shows the position along the fragment. Because the library is prepared of single-stranded DNA, we observe the patterns of C to T on both 3’ and 5’ directions.


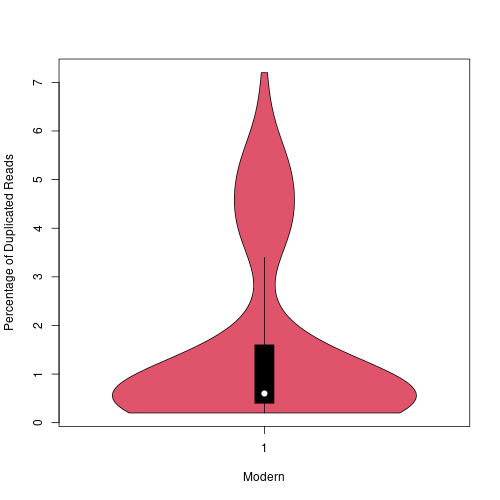

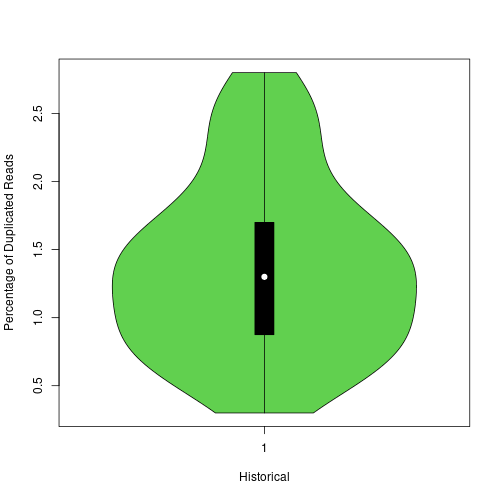


## Figure S17. Distribution of percentage of duplicated reads.

Panels show a violin plot for the distribution of duplicated reads for historical and modern samples, respectively. Y-axis is the percentage of duplicated reads and the width of the violin represents the distribution of fastq files with that percentage of duplicated reads. Values were extracted from the multiQC reports of the raw fastq files for each group.


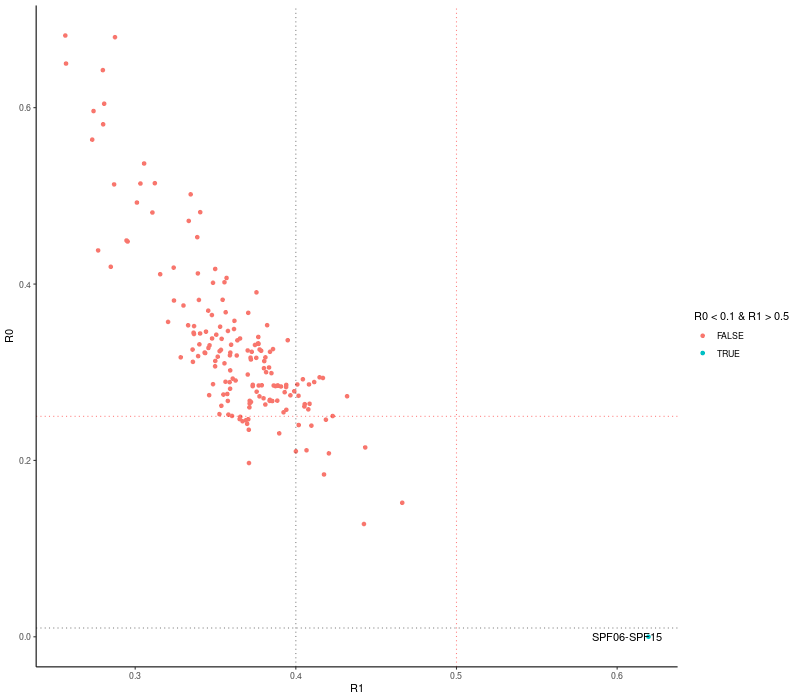

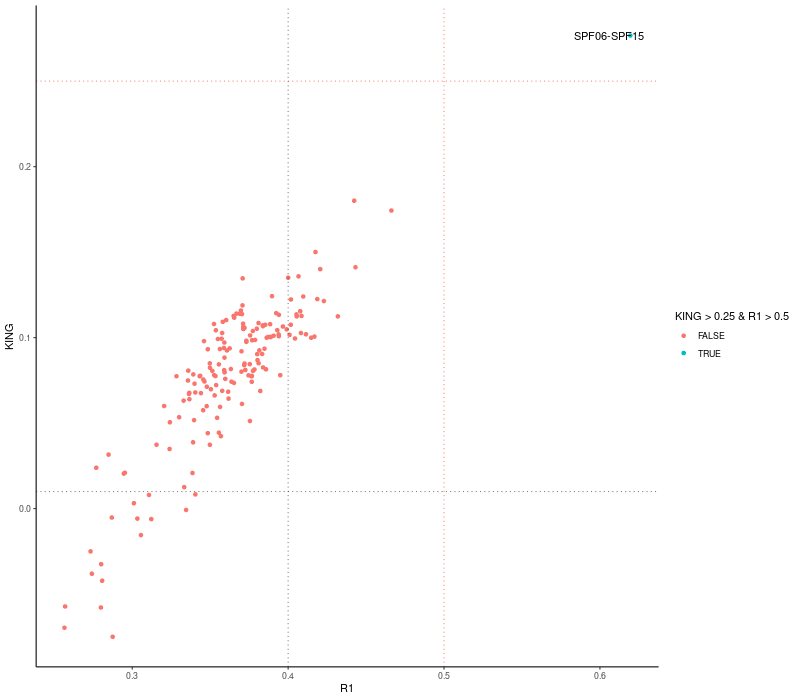


## Figure S18 Genetic relatedness in the modern samples.

We used the method presented by [(Waples et al. 2019)](https://paperpile.com/c/8Rwn1d/CXyjM) which does not rely on allelic frequencies and is suitable for low-coverage data. This method assesses relatedness by comparing two combinations of three kinship statistics making it robust to ascertainment bias: R1 vs R0 and R1 vs KING-robust. We used a low-value threshold for R0 <= 0.1 and high-value thresholds for KING >= 0.25 and R1 >= 0.5 to identify full-sib relationships. We only identified a pair of parent-offspring: SPF06 and SPF15.


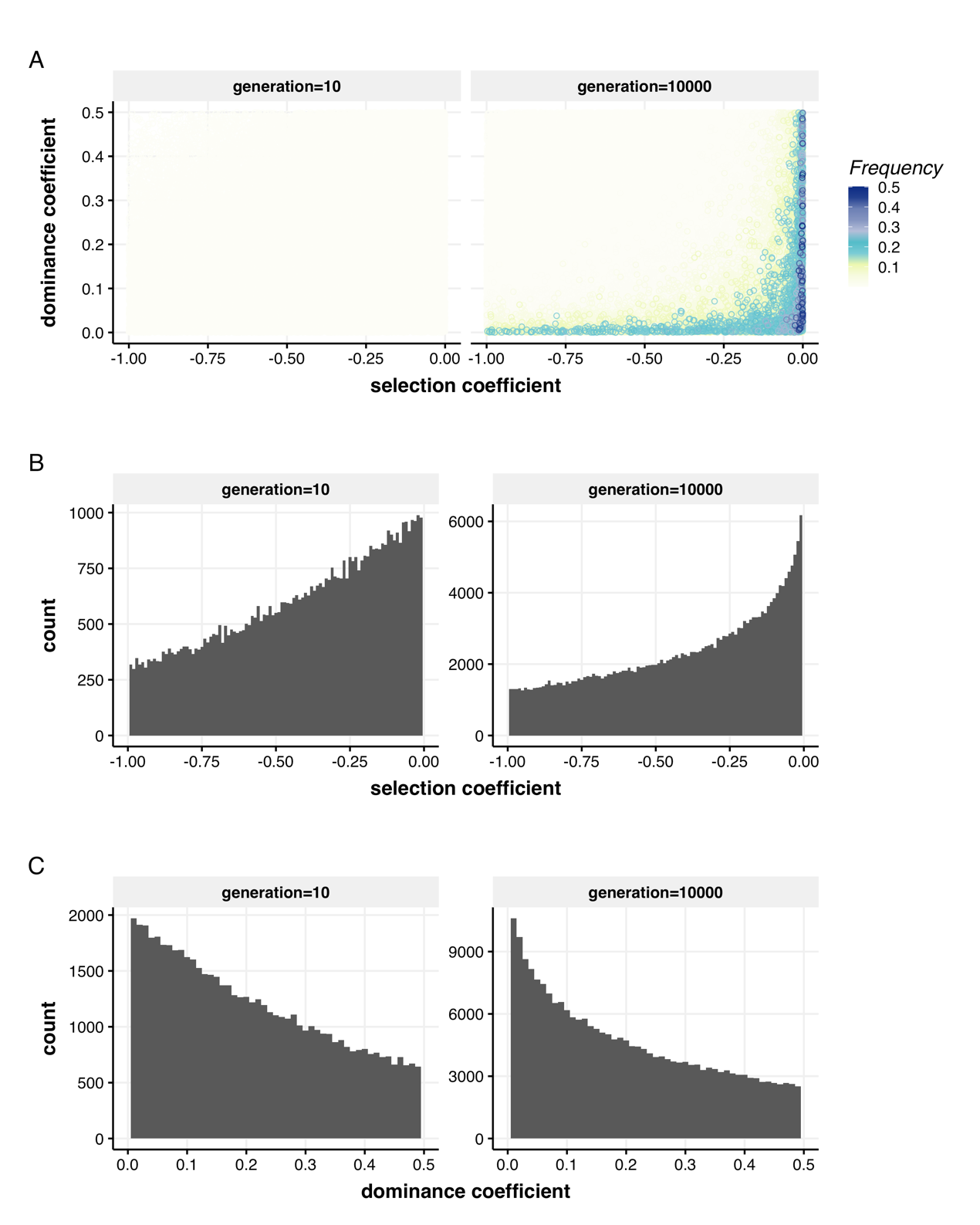


## Figure S19 Distribution of fitness effects (DFE) simulations.

We simulated a large (Ne=20000) population that draws deleterious mutations selection (s) and dominance (h) coefficient values from uniform distributions (-1<s<0 and 0.5<h< 0), allowing any combination of s and h to occur. (A) In the early stages of the simulation, low-frequency mutations with any combination of s and h can be observed. As time progresses, the population approaches a mutation-drift-selection equilibrium, and the expected DFE gamma distribution emerges. Natural selection rapidly purges highly deleterious mutations with high dominance coefficients. However, highly recessive (h<<0.5) mutations and mutations with small selection coefficients (|s|<0.01) can reach appreciable frequencies. Thus, (nearly) recessive highly deleterious mutations can only be maintained in the ancestral population in heterozygous form. We randomly sampled from the resulting simulated DFE (generation=40000) to parametrise our simulations (see Fig S19). (B) A histogram count of selection coefficients shows that already early in the simulation at generation 10 selection prevents the accumulation of high-s mutations, as time progresses this effect of natural selection becomes stronger. (C) A histogram count of selection coefficients shows that already early in the simulation at generation 10 selection prevents the accumulation of high-h mutations, as time progresses this effect of natural selection acting against additive mutations becomes stronger, particularly for the high-s categories (panel A).

**
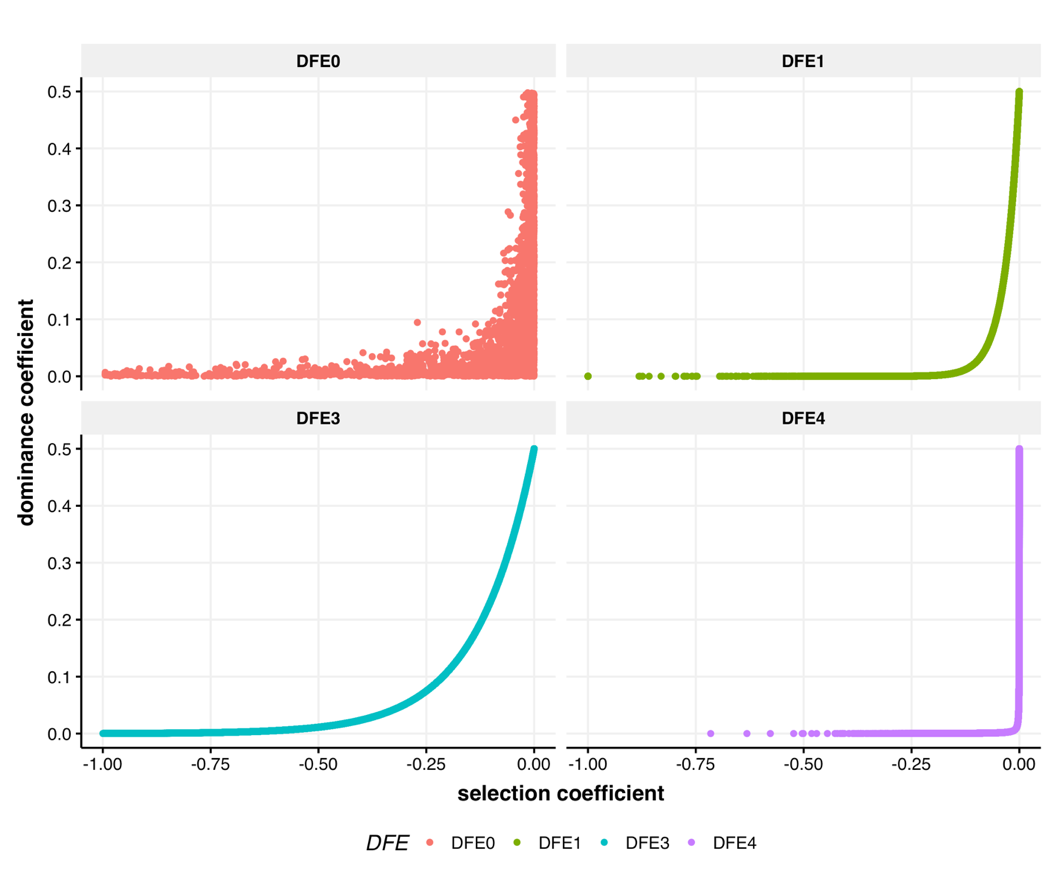
**

## Figure S20 Alternative distributions of fitness effects (DFE).

The resulting DFE from our simulations (DFE0) and alternative DFEs previously used in the literature (DFE1: Kardos et al. 2021; DFE3= Pérez-Pereira et al. 2022; and DFE4: Kyriazis et al. 2021). For DFE0 we draw deleterious mutations selection (s) and dominance (h) coefficient values from uniform distributions (-1<s<0 and 0.5<h< 0), allowing any combination of s and h to occur. For DFE1 we draw s from a gamma distribution mean = -0.05 and shape 0.5 and converted 5% of mutation to be lethal. For DFE3 we draw s from a gamma distribution mean = -0.2 and shape 0.33. For DFE4 we draw s from a gamma distribution mean = -0.013 and shape 0.186.


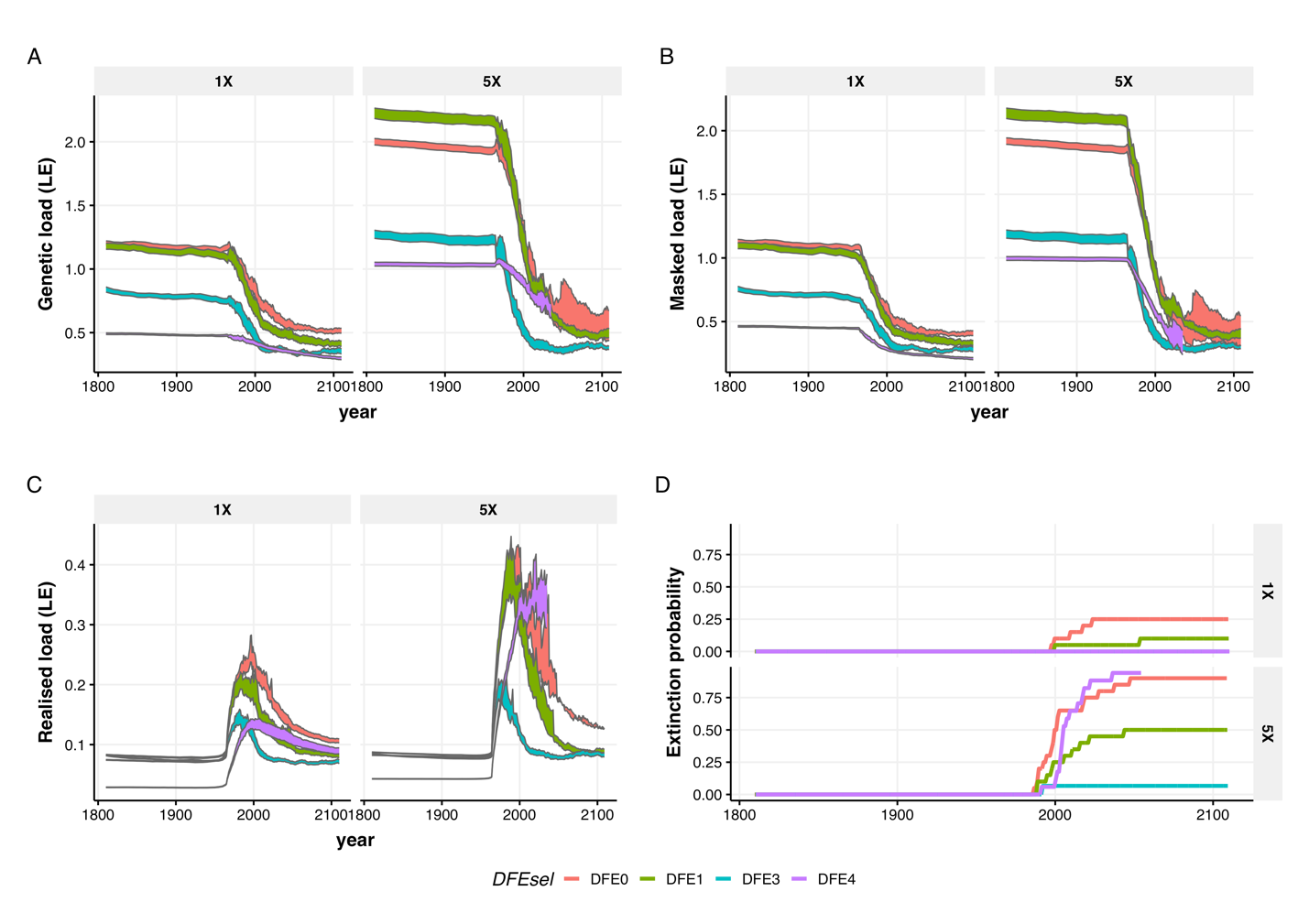


## Figure S21 Effect of alternative distributions of fitness effects (DFE) on the dynamics of the genetic load and extinction risk.

We repeated our simulation using alternative DFEs (DFE0: our simulation (see Fig SXX); DFE1: Kardos et al. 2021; DFE3= Pérez-Pereira et al. 2022; and DFE4: Kyriazis et al. 2021). (A) (Total) genetic load through time measured as lethal equivalents (LE). (B) Masked genetic load through time measured as lethal equivalents (LE). (C) Realised genetic load through time measured as lethal equivalents (LE). Genetic load metrics were calculated following Bertorelle et al 2021. (D) Extinction probability measured as the proportion of replicates that went extinct. The expected overall dynamics of purging of genetic load and conversion of masked to realised load hold true for all the alternative DFEs. Likewise, our prediction of higher extinction risk for a larger ancestral population size (1X vs 5X, see main text) also hold true, validating our findings in the main text. However, the extinction probability differs markedly depending on the DFE, overall, the more recessive the DFE is, the higher the extinction risk. This is an interesting observation worth investigating more deeply, but it falls outside of the scope of this paper.


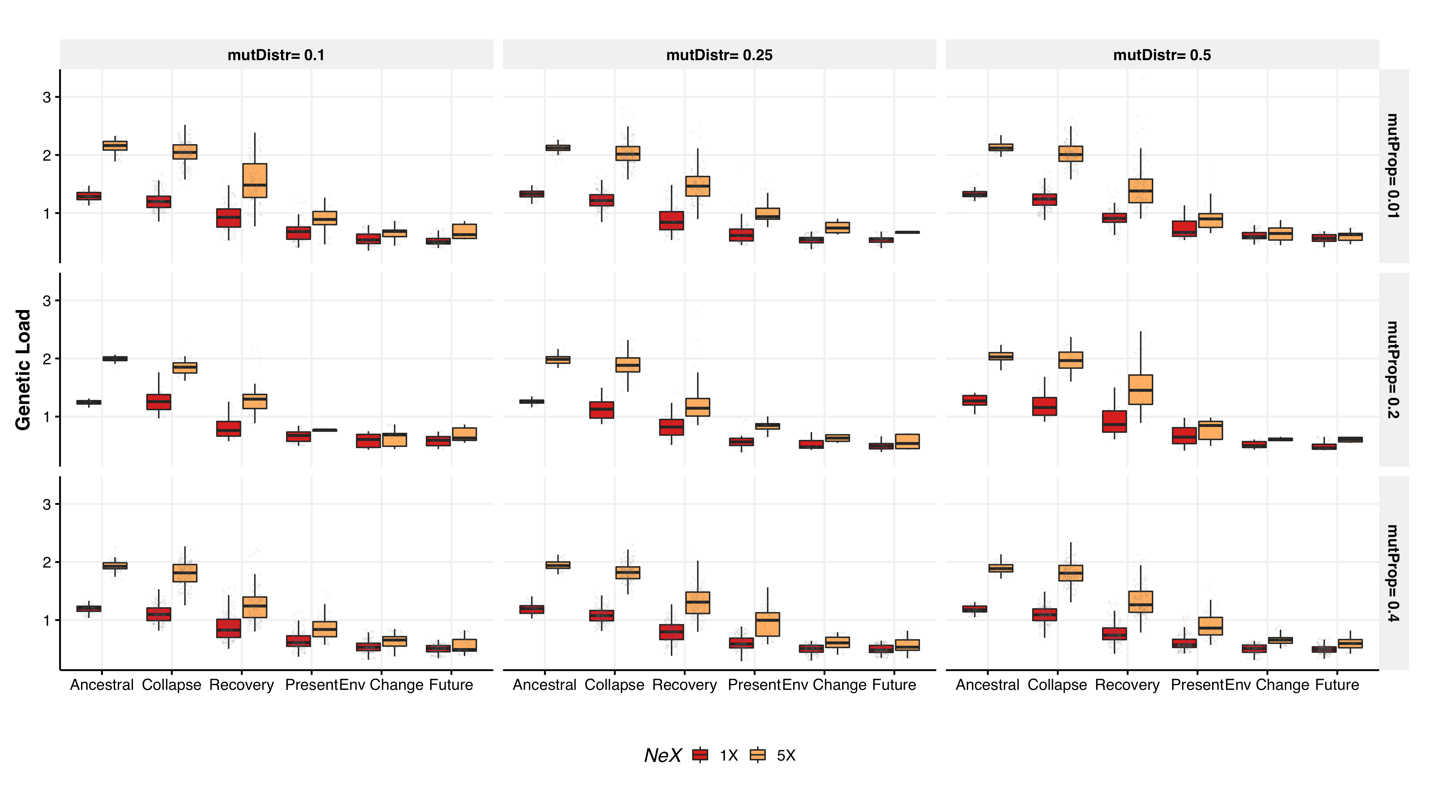


## Figure S22 Parameter test of forward simulations for the (Total) genetic load.

The 1X trend (red) represents the known trajectory and the alternative scenario represents a 5X (yellow) larger ancestral population size. The panels represent alternative parameters. The parameters *mutDistr* is the range of the uniform distribution from which genotype values (z) were drawn for the polygenic trait (-01-0.1, -0.25-0.25 or -0.5-0.5). The parameter *mutProp* is the relative proportion of mutation contributing to the polygenic trait relative to those contributing to the unconditional genetic load.


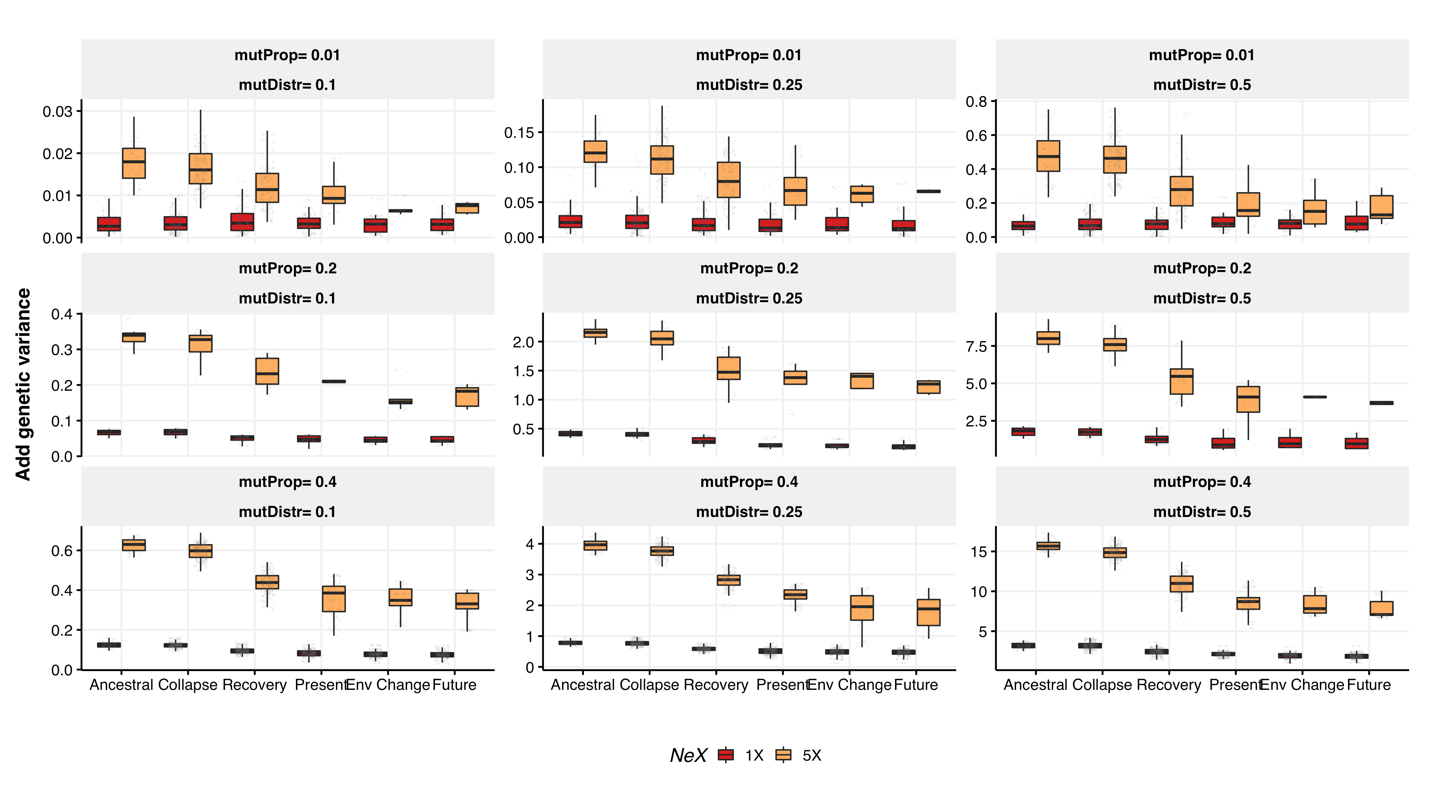


## Figure S23 Parameter test of forward simulations for the additive genetic variance in the quantitative trait (Va).

The 1X trend (red) represents the known trajectory and the alternative scenario represents a 5X (yellow) larger ancestral population size. The panels represent alternative parameters. The parameters *mutDistr* is the range of the uniform distribution from which genotype values (z) were drawn for the polygenic trait (-01-0.1, -0.25-0.25 or -0.5-0.5). The parameter *mutProp* is the relative proportion of mutation contributing to the polygenic trait relative to those contributing to the unconditional genetic load.


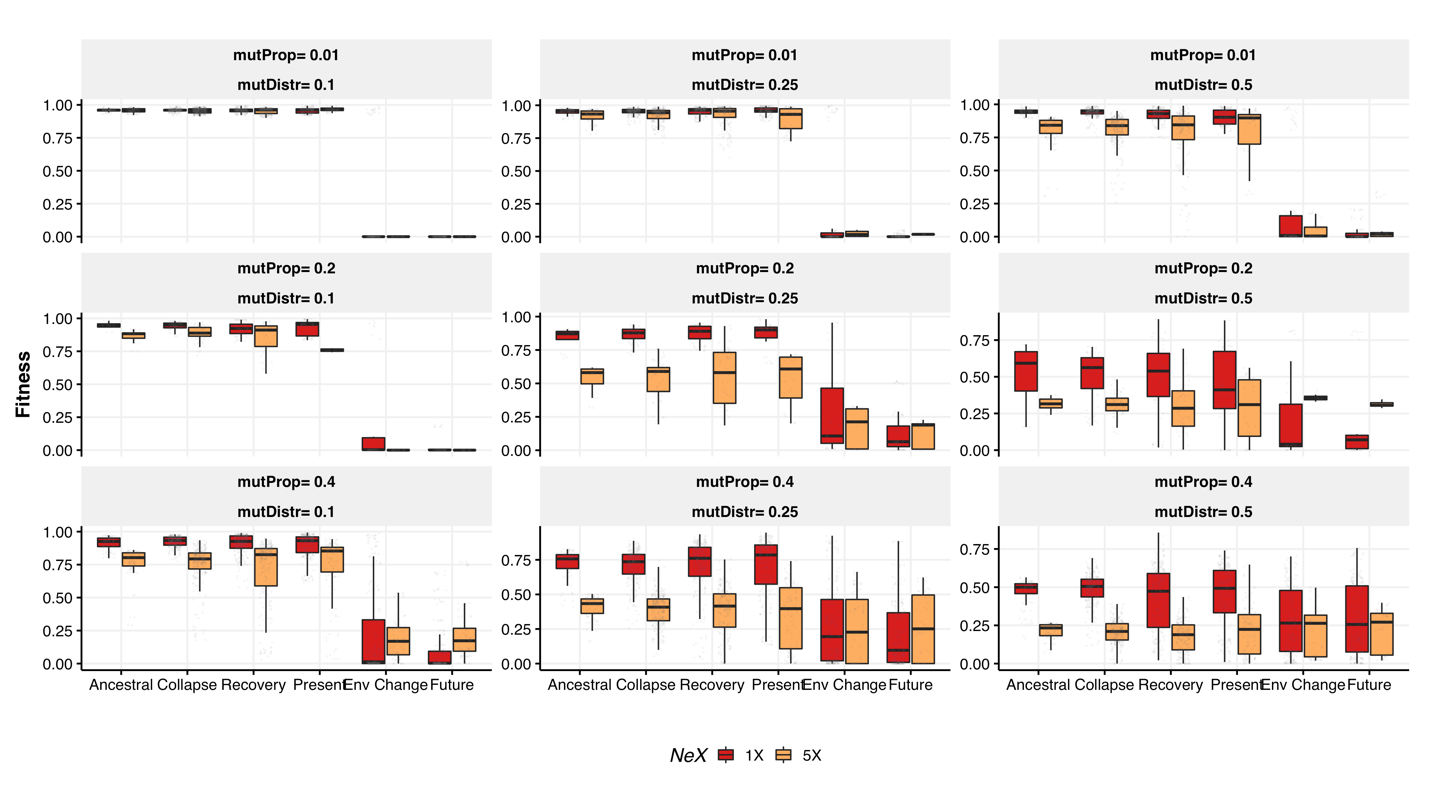


## Figure S24 Parameter test of forward simulations for the fitness effect conferred by the quantitative trait.

The 1X trend (red) represents the known trajectory and the alternative scenario represents a 5X (yellow) larger ancestral population size. The panels represent alternative parameters. The parameters *mutDistr* is the range of the uniform distribution from which genotype values (z) were drawn for the polygenic trait (-01-0.1, -0.25-0.25 or -0.5-0.5). The parameter *mutProp* is the relative proportion of mutation contributing to the polygenic trait relative to those contributing to the unconditional genetic load.

## References

[Backström N, Forstmeier W, Schielzeth H, Mellenius H, Nam K, Bolund E, Webster MT, Ost T, Schneider M, Kempenaers B, et al. 2010. The recombination landscape of the zebra finch Taeniopygia guttata genome. *Genome Res.* 20:485–495.](http://paperpile.com/b/8Rwn1d/NfThE)

[Bellinger MR, Johnson JA, Toepfer J, Dunn P. 2003. Loss of genetic variation in greater prairie chickens following a population bottleneck in Wisconsin, U.s.a. *Conserv. Biol.* 17:717–724.](http://paperpile.com/b/8Rwn1d/iWXqL)

[Cavill EL, Gopalakrishnan S, Puetz LC, Ribeiro ÂM, Mak SST, da Fonseca RR, Pacheco G, Dunlop B, Accouche W, Shah N, et al. 2022. Conservation genomics of the endangered Seychelles Magpie‐Robin ( *Copsychus sechellarum* ): a unique insight into the history of a precious endemic bird. *Ibis*  164:396–410.](http://paperpile.com/b/8Rwn1d/5KAqL)

[Chen N, Van Hout CV, Gottipati S, Clark AG. 2014. Using Mendelian inheritance to improve high-throughput SNP discovery. *Genetics* 198:847–857.](http://paperpile.com/b/8Rwn1d/hWmvz)

[Dutoit L, Burri R, Nater A, Mugal CF, Ellegren H. 2017. Genomic distribution and estimation of nucleotide diversity in natural populations: perspectives from the collared flycatcher (Ficedula albicollis) genome. *Mol. Ecol. Resour.* 17:586–597.](http://paperpile.com/b/8Rwn1d/07dj5)

[Feng S, Fang Q, Barnett R, Li C, Han S, Kuhlwilm M, Zhou L, Pan H, Deng Y, Chen G, et al. 2019. The Genomic Footprints of the Fall and Recovery of the Crested Ibis. *Curr. Biol.* 29:340–349.e7.](http://paperpile.com/b/8Rwn1d/8Al2)

Feng, S., Stiller, J., Deng, Y., Armstrong, J., Fang, Q. I., Reeve, A. H., ... & Zhang, G. (2020). Dense sampling of bird diversity increases power of comparative genomics. Nature, 587(7833), 252-257.

[Gopalakrishnan S, Ebenesersdóttir SS, Lundstrøm IKC, Turner-Walker G, Moore KHS, Luisi P, Margaryan A, Martin MD, Ellegaard MR, Magnússon ÓÞ, et al. 2022. The population genomic legacy of the second plague pandemic. *Curr. Biol.* [Internet]. Available from:](http://paperpile.com/b/8Rwn1d/TBj1K) <http://dx.doi.org/10.1016/j.cub.2022.09.023>

Habic, A., Mattick, J. S., Calin, G. A., Krese, R., Konc, J., & Kunej, T. (2019). Genetic variations of ultraconserved elements in the human genome. OMICS: A Journal of Integrative Biology, 23(11), 549-559.

[Kawakami T, Mugal CF, Suh A, Nater A, Burri R, Smeds L, Ellegren H. 2017. Whole-genome patterns of linkage disequilibrium across flycatcher populations clarify the causes and consequences of fine-scale recombination rate variation in birds. *Mol. Ecol.* 26:4158–4172.](http://paperpile.com/b/8Rwn1d/krHXE)

Kardos M, Armstrong EE, Fitzpatrick SW, Hauser S, Hedrick PW, Miller JM, Tallmon DA, Funk WC. 2021. The crucial role of genome-wide genetic variation in conservation. *Proc. Natl. Acad. Sci. U. S. A.* [Internet] 118. Available from: http://dx.doi.org/10.1073/pnas.2104642118

[Korneliussen TS, Albrechtsen A, Nielsen R. 2014. ANGSD: Analysis of Next Generation Sequencing Data. *BMC Bioinformatics* 15:356.](http://paperpile.com/b/8Rwn1d/ZLTa9)

[Korneliussen TS, Moltke I, Albrechtsen A, Nielsen R. 2013. Calculation of Tajima’s D and other neutrality test statistics from low depth next-generation sequencing data. *BMC Bioinformatics* [Internet] 14. Available from:](http://paperpile.com/b/8Rwn1d/lk49y) <http://dx.doi.org/10.1186/1471-2105-14-289>

Kyriazis CC, Wayne RK, Lohmueller KE. 2021. Strongly deleterious mutations are a primary determinant of extinction risk due to inbreeding depression. *Evol Lett* 5:33–47.

[Lawson LP, Fessl B, Hernán Vargas F, Farrington HL, Francesca Cunninghame H, Mueller JC, Nemeth E, Christian Sevilla P, Petren K. 2017. Slow motion extinction: inbreeding, introgression, and loss in the critically endangered mangrove finch (Camarhynchus heliobates). *Conserv. Genet.* 18:159–170.](http://paperpile.com/b/8Rwn1d/lznvx)

[Li S, Li B, Cheng C, Xiong Z, Liu Q, Lai J, Carey HV, Zhang Q, Zheng H, Wei S, et al. 2014. Genomic signatures of near-extinction and rebirth of the crested ibis and other endangered bird species. *Genome Biol.* 15:557.](http://paperpile.com/b/8Rwn1d/hMofM)

[Liu S, Westbury MV, Dussex N, Mitchell KJ, Sinding M-HS, Heintzman PD, Duchêne DA, Kapp JD, von Seth J, Heiniger H, et al. 2021. Ancient and modern genomes unravel the evolutionary history of the rhinoceros family. *Cell* 184:4874–4885.e16.](http://paperpile.com/b/8Rwn1d/mRnG8)

Pérez-Pereira N, Pouso R, Rus A, Vilas A, López-Cortegano E, García-Dorado A, Quesada H, Caballero A. 2021. Long-term exhaustion of the inbreeding load in Drosophila melanogaster. *Heredity* 127:373–383.

[Perrier C, Delahaie B, Charmantier A. 2018. Heritability estimates from genomewide relatedness matrices in wild populations: Application to a passerine, using a small sample size. *Mol. Ecol. Resour.* 18:838–853.](http://paperpile.com/b/8Rwn1d/RECR5)

[Perrier C, Lozano del Campo A, Szulkin M, Demeyrier V, Gregoire A, Charmantier A. 2018. Great tits and the city: Distribution of genomic diversity and gene-environment associations along an urbanization gradient. *Evol. Appl.* 11:593–613.](http://paperpile.com/b/8Rwn1d/mWdT0)

[Robinson JA, Bowie RCK, Dudchenko O, Aiden EL, Hendrickson SL, Steiner CC, Ryder OA, Mindell DP, Wall JD. 2021. Genome-wide diversity in the California condor tracks its prehistoric abundance and decline. *Curr. Biol.* 31:2939–2946.e5.](http://paperpile.com/b/8Rwn1d/h8MxV)

[Robledo-Ruiz DA, Gan HM, Kaur P, Dudchenko O, Weisz D, Khan R, Lieberman Aiden E, Osipova E, Hiller M, Morales HE, et al. 2022. Chromosome-length genome assembly and linkage map of a critically endangered Australian bird: the helmeted honeyeater. *Gigascience* [Internet] 11. Available from:](http://paperpile.com/b/8Rwn1d/YkuKu) <http://dx.doi.org/10.1093/gigascience/giac025>

[Segelbacher G, Strand TM, Quintela M, Axelsson T, Jansman HAH, Koelewijn H-P, Höglund J. 2014. Analyses of historical and current populations of black grouse in Central Europe reveal strong effects of genetic drift and loss of genetic diversity. *Conserv. Genet.* 15:1183–1195.](http://paperpile.com/b/8Rwn1d/O4ws)

[von Seth J, van der Valk T, Lord E, Sigeman H, Olsen R-A, Knapp M, Kardailsky O, Robertson F, Hale M, Houston D, et al. 2022. Genomic trajectories of a near-extinction event in the Chatham Island black robin. *BMC Genomics* 23:747.](http://paperpile.com/b/8Rwn1d/popcy)

[Shultz AJ, Baker AJ, Hill GE, Nolan PM, Edwards SV. 2016. SNPs across time and space: population genomic signatures of founder events and epizootics in the House Finch (Haemorhous mexicanus). *Ecol. Evol.* 6:7475–7489.](http://paperpile.com/b/8Rwn1d/uGdbr)

[Taylor SS, Jamieson IG, Wallis GP. 2007. Historic and contemporary levels of genetic variation in two New Zealand passerines with different histories of decline. *J. Evol. Biol.* 20:2035–2047.](http://paperpile.com/b/8Rwn1d/pPfnM)

[Thompson EA. 2013. Identity by Descent: Variation in Meiosis, Across Genomes, and in Populations. *Genetics* 194:301–326.](http://paperpile.com/b/8Rwn1d/npGd9)

[de Villemereuil P, Rutschmann A, Lee KD, Ewen JG, Brekke P, Santure AW. 2019. Little Adaptive Potential in a Threatened Passerine Bird. *Curr. Biol.* 29:889–894.e3.](http://paperpile.com/b/8Rwn1d/CF7rL)

[Waples RK, Albrechtsen A, Moltke I. 2019. Allele frequency-free inference of close familial relationships from genotypes or low‐depth sequencing data. *Molecular Ecology* [Internet] 28:35–48. Available from:](http://paperpile.com/b/8Rwn1d/CXyjM) <http://dx.doi.org/10.1111/mec.14954>
